# Characterization and genome analysis of six novel *Vibrio parahaemolyticus* phages associated with acute hepatopancreatic necrosis disease (AHPND)

**DOI:** 10.1016/j.virusres.2022.198973

**Published:** 2022-10-20

**Authors:** Alma Karen Orozco-Ochoa, Jean Pierre González-Gómez, Nohelia Castro-del Campo, Juan Daniel Lira-Morales, Célida Isabel Martínez-Rodríguez, Bruno Gomez-Gil, Cristóbal Chaidez

**Affiliations:** a Centro de Investigación en Alimentación y Desarrollo, A.C. (CIAD), Laboratorio Nacional para la Investigación en Inocuidad Alimentaria (LANIIA), Carretera a Eldorado Km 5.5, Campo El Diez, Culiacán, Sinaloa 80110, México; b Centro de Investigación en Alimentación y Desarrollo, A.C. (CIAD), Unidad Mazatlán en Acuicultura y Manejo Ambiental, Mazatlán, Sinaloa AP 711, México

**Keywords:** Phages, *Vibrio parahaemolyticus*, AHPND, Characterization, Genome analysis

## Abstract

*Vibrio parahaemolyticus* causes acute hepatopancreatic necrosis disease (AHPND) in farmed shrimp. Due to its damage potential, which could be as high as a 100% mortality rate, bacteriophages have emerged as a promising natural control intervention other than antibiotics, yet multiple roadblocks need to be overcome*.* In this study, six bacteriophages isolated from seafood samples, seawater, and estuary water in Sinaloa, Mexico, demonstrated a narrow host range among Mexican AHPND-causing *V. parahaemolyticus*. All bacteriophages are composed of a double-stranded DNA genome with lengths ranging between 43,268 and 57,805 bp. All six phages exhibited latency periods of 10–30 min and burst sizes of 34–168 viral particles per infected cell. The optimal MOI for bacteriophage propagation was 0.01–1. No transfer RNA (tRNA), virulence, or resistance genes were found in either genome, and the life cycle of these phages was classified as virulent by the PhageAI platform. Phylogenetic and comparative genomics analyzes assigned phages M3, C2, M9, and M83 as new species not yet reported within the genus *Maculvirus, Autographiviridae* family. ALK and CHI phages were assigned as new members of a new genus not yet classified within the subfamily *Queuovirinae*. The findings highlight the potential of CHI, ALK, M3, C2, M9, and M83 as promising alternatives against AHPND-causing *V. parahaemolyticus* from Mexico.

## Highlights

• Phages M3, C2, M9, and M83 belong to a new species of the genus of *Maculvirus* and phages ALK and CHI belong to new genus of the *Queuovirinae* subfamily.
• CHI, ALK, M3, C2, M9, and M83 could lyse 50.0% (5/10) of Mexican AHPND-causing *Vibrio parahaemolyticus* strains.
• No transfer RNA (tRNA), virulence, or antibiotic resistance genes were found in phage genomes.
• CHI, ALK, M3, C2, M9, and M83 significantly reduced the growth of Mexican AHPND-causing *Vibrio parahaemolyticus* strains after incubating with MOI of 0.01–1.

## 1 Introduction

*Vibrio parahaemolyticus* is a gram-negative halophilic bacterium, that is typically found in marine habitats worldwide (Thammatinna et al., 2020) and is commonly associated with a major threat to aquaculture and human health. *V. parahaemolyticus* is an important cause of bacterial gastroenteritis in humans due to the ingestion of raw or undercooked fish, shrimp, shellfish, and other seafood (Yang et al., 2020, 2022). In addition, *V. parahaemolyticus* is the main causal agent and is most prevalently detected, causing acute hepatopancreatic necrosis disease (AHPND) in shrimp (González-Gómez et al., 2022; Tran et al., 2013) and economic losses in the shrimp farming industry (Hong et al., 2016; Han et al., 2020), the major activity that sustains the social development of many regions in the world (Alagappan et al., 2010, 2016). AHPND, originally known as early mortality syndrome (EMS), is a newly emerging disease in shrimp (Xiao et al., 2017). AHPND is a highly virulent disease that affects several shrimp species and is characterized by severe atrophy of the hepatopancreas, with a mortality rate of nearly 100% within the first 35 days of the postlarval stage (Hong et al., 2016). The disease is mainly caused by *V. parahaemolyticus*, which harbors the pVA1 plasmid, which secretes PirA- and PirB- like binary toxins that cause deterioration in the hepatopancreas of infected shrimp (Han et al., 2015). Since the first outbreak was reported in China in 2009, AHPND has spread to other Asian countries and Mexico (2013); the disease has caused serious global economic losses estimated up to USD $1 billion annually in the shrimp farming industry (FAO, 2013; Hong et al., 2016; Han et al., 2020; Yang et al., 2020). To date, we know that this disease continues to affect Mexican shrimp production in the Pacific Northwest region of Mexico, but there are no reports of outbreaks. Additionally, there is little regional availability of vibriophages against AHPND to test its effect against strains of local interest.

Thus, to date, antibiotics are used as a common therapeutic agent in shrimp farms to control bacterial diseases (Luu et al., 2021). Another approach to control this disease has focused on using probiotics and chemical intervention (disinfection) (González-Gómez et al., 2022). However, the extensive use of antibiotics has resulted in the development of multidrug-resistant pathogens (Narayanan et al., 2020); this has been particularly the case for *V. parahaemolyticus*, which has a significantly high drug resistance rate, including the antibiotics currently used in aquaculture and even in clinical settings (You et al., 2021). The options for alternative treatments have also had potential side effects on shrimp quality and the environment (Soto-Rodriguez et al., 2015; Kumar et al., 2020). Therefore, safe alternative strategies are needed to control AHPND and prevent the spread of multidrug-resistant bacteria in shrimp aquaculture.

Bacteriophages (phages) have become a promising alternative for bacterial control in aquaculture (Ding et al., 2020) as viruses that infect specific bacteria, and there is an increasing number of studies regarding the application of phage therapy with satisfactory results (Karunasagar et al., 2007; Lomelí-Ortega and Martínez-Díaz, 2014; Chen et al., 2019). The therapeutic use of bacteriophages (phage therapy) has recently emerged as a promising and environmentally friendly alternative that offers attractive options for treating multidrug-resistant bacteria (Altamirano and Barr, 2019), while maintaining food safety (Tan et al., 2021). However, bacteriophages require a deep understanding of their genomic and biological characteristics before their use in phage therapy. They must at a minimum meet suggested regulatory requirements, such as demonstrating strictly lytic activity, confirmed antimicrobial activity against the target pathogen, and not containing virulence or resistance to antibiotic genes in their genome (Young and Gill, 2015; Droubogiannis and Katharios, 2022). To date, few reported phages infect Mexican strains (Makarov et al., 2019; González-Gómez et al., 2022). However, the main effort needed so that phages can be established as an effective control method is to establish a specific and reliable phage library, with at least genome analysis and biological characterization, that can rapidly be phage specific against AHPND-associated *V. parahaemolyticus* strains. It can also help to have multiple phages available that infect the same strain but bind to different receptors for effective disease control by formulating cocktails and thus avoid resistance to phages. Therefore, our isolation and characterization of new phages of Mexican AHPND-associated *V. parahaemolyticus* efforts are vitally important to creating a well-characterized phage library against AHPND-*Vibrio parahaemolyticus* strains available in Mexico, openly available to Mexican shrimp farming, as there are practically no phages with this level of characterization available in the region to combat AHPND. Here, we isolate six novel vibriophages, CHI, ALK, M3, C2, M9, and M83, from seawater, estuarine water, and shellfish samples, which present lytic activity against Mexican AHPND-causing *V. parahaemolyticus* strains. We expect that these vibriophages will be used as a viable alternative to control *V. parahaemolyticus* in aquaculture.

## 2 Materials and methods

### 2.1 Bacterial strains and growth conditions

A total of 10 previously reported AHPND-causing *V. parahaemolyticus* strains isolated in Mexico from 2013 to 2019 (Supplementary Table S9) (Gomez-Gil et al., 2014; Soto-Rodriguez et al., 2015; González-Gómez et al., 2020) were provided by CIAD, AC Mazatlan Unit for Aquaculture and Environmental Management. All strains were cultivated in tryptic soy broth (TSB; Oxoid Ltd, Hants, UK) supplemented with 2.5% NaCl (Jalmek, Nuevo Leon, Mexico) and incubated at 37 °C for 18–24 h. Stock cultures were stored in 20% (v/v) glycerol at -80 °C.

### 2.2 Phage isolation and purification

The presence of bacteriophages was evaluated in estuarine and beach water and seafood from Sinaloa's beaches, including Altata, El Maviri, and Topolobampo Bay (Table 1). Samples were processed by the enrichment method as described previously (Mateus et al., 2014; Thammatinna et al., 2020; González-Gómez et al., 2022) with some modifications. Briefly, to prepare the bacterial culture of solid samples, 5 g of seafood was added to 45 mL of tryptic soy broth supplemented with 2.5% sodium chloride (TSB-NaCl 2.5%) and 1 mL of each overnight bacterial culture. To prepare the bacterial culture of liquid samples, 200 mL of water was added to 200 mL of TSB at a double concentration supplemented with 2.5% sodium chloride (2x TSB-NaCl 2.5%) and 1 mL of each overnight bacterial culture. Then, both were incubated at 30 °C for 18 h at 80 rpm. After incubation, the enriched samples were centrifuged at 10,000 rpm for 10 min at 4 °C (Megafuge16R, Thermo Fisher Scientific Inc., Waltham, Massachusetts, USA), and the supernatants were collected and filtered through a 0.45 μm pore membrane (Pall Corp., NY, USA). The presence/absence of bacteriophages was verified through a spot test. The filtrates that showed the presence of bacteriophages were serially diluted, and 100 μL of diluted supernatant was mixed with 1000 μL of an overnight culture of host bacteria in 3 mL of soft agar (TSB with 0.5% agar), poured on tryptic soy agar (TSA) plates (Oxoid Ltd, Hants, UK), and incubated at 37 °C for 18 h. After incubation, bacteriophage plaques were selected based on size and clarity. Purification of the phages was performed at least three times with SM buffer (50 mM Tris-Cl [1 M, pH 7.5], 100 mM NaCl, 8 mM MgSO_4_·7H_2_O). Finally, purified and targeted phages were stored at 4 °C in TSB for further studies and at -80 °C in 20% (v/v) glycerol.

**Table 1:** Summary of the characteristics of the different phages isolated in Sinaloa, Mexico.

Phage strain ID	Isolation			Host range (Spot test) EOP^a^										Plaque diameter (mm)^b^
	Source	Locality	Geographic coordinate	M0605	M0607	M0802	M0803	M0904	M0905	M2401	M2411	M2413	M2415	
CHI	Chinese snail	Los Mochis, Sinaloa, Mexico	108°58′46.52′′W 25°47′31.99′′N	-	-	-	-	++ (0.75)H	-	++ (0.74)H	++ (1.0)H	-	-	2
M3	Seawater	El Maviri, Sinaloa, Mexico	25°34′55.7"N 109°07′05.1"W	-	-	-	-	-	-	-	-	++ (0.43)M	++ (1.0)H	1
ALK	Chocolate Clam	Los Mochis, Sinaloa, Mexico	108°58′46.52′′W 25°47′31.99′′N	-	-	-	-	++ (0.43)M	-	++ (0.46)M	++ (1.0)H	+ (0.01)L	+ (0.02)L	2
M83	Estuary water	El Maviri, Sinaloa, Mexico	25°36′47.5"N 109°03′55.3"W	-	-	-	-	+ (0.02)L	-	-	-	++ (1.0)H	+ (0.11)M	2
M9	Estuary water	Topolobampo, Sinaloa, Mexico	25°36′23.2"N 109°03′31.5"W	-	-	-	-	-	-	-	-	++ (1.0)H	+ (0.02)L	1
C2	Shrimp	Los Mochis, Sinaloa, Mexico	25°47′26.7"N 108°59′59.8"W	-	-	-	-	-	-	-	-	++ (1.0)H	++ (0.9)H	1

a Efficiency of plating (EOP) was classified as “H” high efficiency (EOP ≥0.5), “M” medium efficiency (0.1≤EOP≺0.5), “L” low efficiency (0.001≺EOP≺0.1) and “I” ineffective (EOP≤0.001); Host range results were classified as clear (++) plaques, positive opaque plaques (+), and no lysis (-).

b Plaque diameter obtained from the double agar technique against their respective host.

### 2.3 Phage host range and efficiency of plating (EOP)

The host range of phages CHI, ALK, M3, C2, M9, and M83, was determined using both the spot test and the efficiency of plating (EOP) as described previously with some modifications (Hu et al., 2021). Briefly, 10 μL of purified phage suspensions (10^7^-10^8^ PFU/mL) were spotted on freshly inoculated *V. parahaemolyticus* lawns on TSA plates and left to dry at room temperature before incubation for 18 h at 37 °C. The results were classified based on the clarity of the spot and divided into three categories: clear lysis plaques (++), positive opaque lysis plates (+), and no lysis (-). All *V. parahaemolyticus* isolates sensitive to the phages CHI, ALK, M3, C2, M9, and M83 in the spot test were selected to determine the EOP by the double-agar layer method (Cao et al., 2021). The EOP values were calculated by dividing the average of lysis plaques produced in each susceptible strain by the number of plaques produced on the best host and then ranked as “high efficiency” (EOP ≥ 0.5), “medium efficiency” (0.1 ≤ EOP < 0.5), “low efficiency” (0.001 < EOP < 0.1) or “inefficient” (EOP ≤ 0.001).

### 2.4 One-step growth curve

The one-step growth curves were performed according to Kropinski et al. (2018), with minor modifications. Briefly, 1 mL of the bacterial solution was diluted (10^8^ CFU/mL) and centrifuged at 8000 *x g* for 5 min. The pellet was resuspended in 1 mL of SM buffer. The phage (100 µL) was added at MOI= 0.1 and incubated for 15 min at 37 °C. After, the mixture was centrifuged at 12,000 *x g* for 2 min to remove free phage, and the pellet was suspended in 1000 μL of TSB+2.5%. Thereafter, 1 mL of this mixture was added to 10 mL of TSB+2.5% NaCl. Finally, two samples were collected at 0 min, and one of them was added to 50 μL of chloroform. The mixture was incubated at 37 °C with shaking (200 rpm), and samples were collected every 10 min over a period of 80 min and titrated using the double-agar layer method. The experiment was repeated three times. The burst size was calculated as the ratio of the final count of liberated phage particles to the initial count of infected bacterial cells divided by the number of infected cells (phage titer at 0 min – phage titer at 0 min with chloroform).

### 2.5 Phage thermal and pH stability test

To investigate the stability under different temperatures and pH values of isolated phages, we followed a previously described method (Stalin and Srinivasan, 2017) with some minor changes. In brief, 100 μL of phages was suspended in 900 μL of SM buffer (pH 7.5) and kept at different temperatures (30, 40, 50, 60, and 70 °C). After 1 h of incubation, the phage titer was evaluated by the double-layer agar method. To determine the stability of the phages at different pH levels, phage stock was added to the SM buffer. To evaluate and measure the pH stability, 100 μL of phage suspension was added to 900 μL of SM buffer with a pH of 2, 3, 5, 7, 9, 11, and 12, and then incubated at 37 °C for 1 h. The pH value of SM buffer was adjusted by adding HCl and NaOH solutions. Similarly, the titer of each surviving phage was enumerated using the double-layer agar method, as described previously.

### 2.6 Phage survival stability at different storage temperatures

To determine the stability of phages under different storage temperatures, an experiment was performed with a previously described method with some changes (Huan et al., 2019). Briefly, 100 μL of phage suspension (10^7^ PFU/mL) was suspended in 900 μL of SM buffer (pH 7.5). It was stored at different temperatures (-80, 25, and 4 °C) for one month. After the incubation period, the phage titer was evaluated by the double-layer agar method.

### 2.7 *In vitro* bacteriolytic activity test

The *in vitro* bacteriolytic activity of phages CHI, ALK, M3, C2, M9, and M83 was determined using UV‒vis spectroscopy. Additionally, the bacterial strains used in the experiment were Mexican AHPND-causing *V. parahaemolyticus* strains M0904 and M2413 as described previously (Ding et al., 2020) with minor modifications. Briefly, the bacterial solution was diluted to 1 × 10^8^ CFU (1 mL) and added to 50 mL fresh TSB+2.5% NaCl. The phage evaluated was mixed with the *V. parahaemolyticus* culture at different MOIs of 1, 0.1, and 0.01 and a control group without phage (0.00), followed by incubation at 37 °C for 24 h. *V. parahaemolyticus* culture by itself also served as a positive control. The optical density (OD_600nm_) of the culture was measured at the indicated time points (0, 1, 2, 3, 4, 5, 6, 8, and 24 h) on a UV‒vis spectrophotometer.

### 2.8 Genomic DNA extraction and sequencing

Phage genomic DNA was extracted using the phenol‒chloroform method (Sambrook and Russell, 2006). Briefly, 1 mL of phage suspension was transferred to a 1.5 mL microtube and treated with 10 μL of DNase I/RNase A (10 mg/mL) at 37 °C for 30 min, followed by treatment with 50 μL SDS (10%), 40 μL EDTA (0.5 M), and 2.5 μL proteinase K (20 mg/mL) and incubation at 56 °C for 2 h. An equal volume of phenol was added, mixed, and centrifuged at 3,500 *x g* for 10 min. The aqueous layer was transferred and mixed with an equal volume of phenol‒chloroform 1:1 (v/v) and centrifuged three times at 3,500 *x g* for 10 min at 25 °C. At the end of the centrifugation, the aqueous layer was collected and mixed with 200 μL sodium acetate 3 M and ethyl alcohol until the tube was filled and stored at -20 °C overnight. At the end of the incubation, the mixture was centrifuged at 15,000 *x g* for 30 min, and the DNA pellet was washed with 70% ethyl alcohol three times. Finally, the DNA pellet was air-dried, resuspended in 20 μL of nuclease-free water, and stored at -20 °C. The DNA was quantitated using a NanoDrop 2000c spectrometer (Thermo Scientific, Wilmington, USA), and the quality of the DNA was assessed by electrophoresis.

The DNA libraries were prepared using the Nextera XT Library Preparation Kit (Illumina, San Diego, CA, USA) according to the manufacturer's instructions, and genome sequencing was performed with the Illumina MiniSeq platform (2×150 bp paired-end protocol, 300 cycles). Raw reads were paired and trimmed by fastp v0.23.0 and de novo assembled using SPAdes v3.12.0 (Bankevich et al., 2012; Chen et al., 2018; Wick, 2021).

### 2.9 Bioinformatics analyzes

After sequence assembly, blastn was used to compare the whole genome sequences of phages with those in the NCBI database, and the phages with the closest sequences in GenBank were identified. Contigs were annotated using the phannotate algorithm in PATRIC v.3.6.12 (https://www.patricbrc.org/app/Annotation). Phages closely related to those isolated here were examined using an online blastn in the GenBank database. The putative transfer RNA (tRNA) encoding genes were predicted using tRNAscan-SE (Lowe and Chan, 2016). The presence of resistance genes and virulence genes was examined against the NCBI, CARD, ARG-ANNOT, Resfinder, MEGARES, PlasmidFinder, and VFDB databases through ABRicate v.0.8.13 (https://github.com/tseemann/abricate). The AI-driven software platform PhageAI v0.10.0 (https://phage.ai/) was used to classify the phage lifestyle.

The putative open reading frames (ORFs) were annotated using the protein basic local alignment search tool (blastp) of the NCBI server (https://blast.ncbi.nlm.nih.gov/Blast.cgi) based on a search of the Non-Redundant Protein Database of NCBI, with the score set at > 50 and an e-value of <1.0 × 10^–3^. The ORF functions were curated using Geneious v9.1.8. For the phylogenetic analysis, protein sequences were aligned using PATRIC v.3.6.12. Based on the alignments, complete phage genomes were assessed using the VICTOR web tool (https://ggdc.dsmz.de/victor.php) to determine the phylogenetic relationships (Meier-Kolthoff and Goker, 2017) and included phylogenomic trees inferred using the Genome-BLAST Distance Phylogeny method (GBDP). The outputs in Newick format were used to create a phylogenetic tree by iTOL (https://itol.embl.de). Finally, the complete genomes were used to calculate intergenomic similarities among viral genomes through the VIRIDIC web tool (Moraru et al., 2020) with blastn default settings. The Artemis Comparison Tool (ACT) was used to compare other phages in GenBank and identify feature variations between the genomes of phages, with homology assessed using blastn (Carver et al., 2005). Furthermore, a global genome comparison map among available phage data in NCBI was visualized by the Easyfig v2.2.2 visualization tool (Sullivan et al., 2011).

### 2.10 Statistical analysis

Experiments were performed in triplicate, and the results are expressed as the mean ± standard deviation (SD). Statistical analysis was performed using Minitab® 19, Minitab Statistical Software (Minitab, LLC.). Significant differences were analyzed using one-way analysis of variance (ANOVA) with Tukey's test and least significance difference (LSD). Statistical significance was set at *P* < 0.05.

## 3 Results

### 3.1 Isolation and general features of bacteriophages

**Fig. 1:**
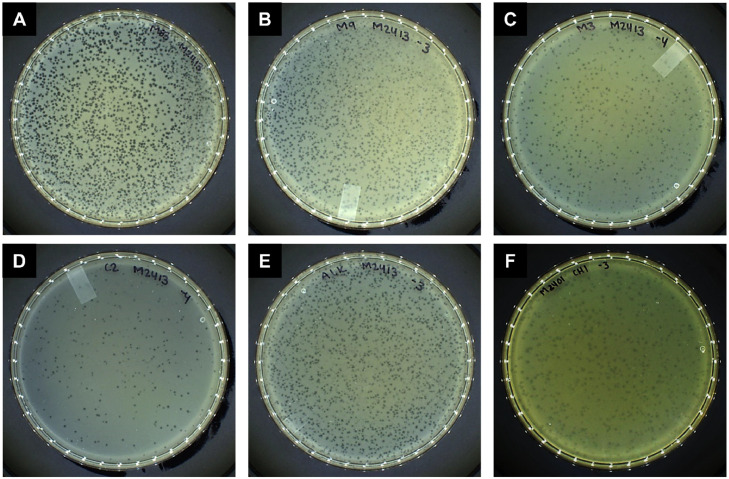
Morphology of plaques formed on a double-layered agar plate by (A) M83, (B) M9, (C) M3, (D) C2, (E) ALK, and (F) CHI.

Twenty-eight seawater, estuary water, and seafood samples were collected and tested for the presence of phages with lytic activity against Mexican AHPND-causing *V. parahaemolyticus* strains. Six phages were successfully purified using the double-agar layer method and designated vB_VpaS_CHI, vB_VpaS_ALK, vB_VpaP_M3, vB_VpaP_C2, vB_VpaP_M9, and vB_VpaP_M83, according to the Bacterial and Archaeal Viruses Subcommittee (BAVS) of the International Committee on Taxonomy of Virus (ICTV) recommendation (Adriaenssens and Brister, 2017). Overall, the lysis plaques observed were clear, uniform, with a circular shape, and average size, approximately 1-2 mm in diameter (Fig. 1).

### 3.2 Host range of phage and efficiency of plating (EOP)

A total of 10 Mexican AHPND-causing *V. parahaemolyticus* strains were tested for a host of phages. In five of these Mexican AHPND-causing *V. parahaemolyticus* isolates, accounting for 5 of 10, the phages CHI, ALK, M3, C2, M9, and M83 formed transparent to turbid plaques. The efficiency of plating was classified as high (EOP ≥ 0.5) or medium (0.1 ≤ EOP < 0.5) (Table 1).

### 3.3 One-step growth curve

**Fig. 2:**
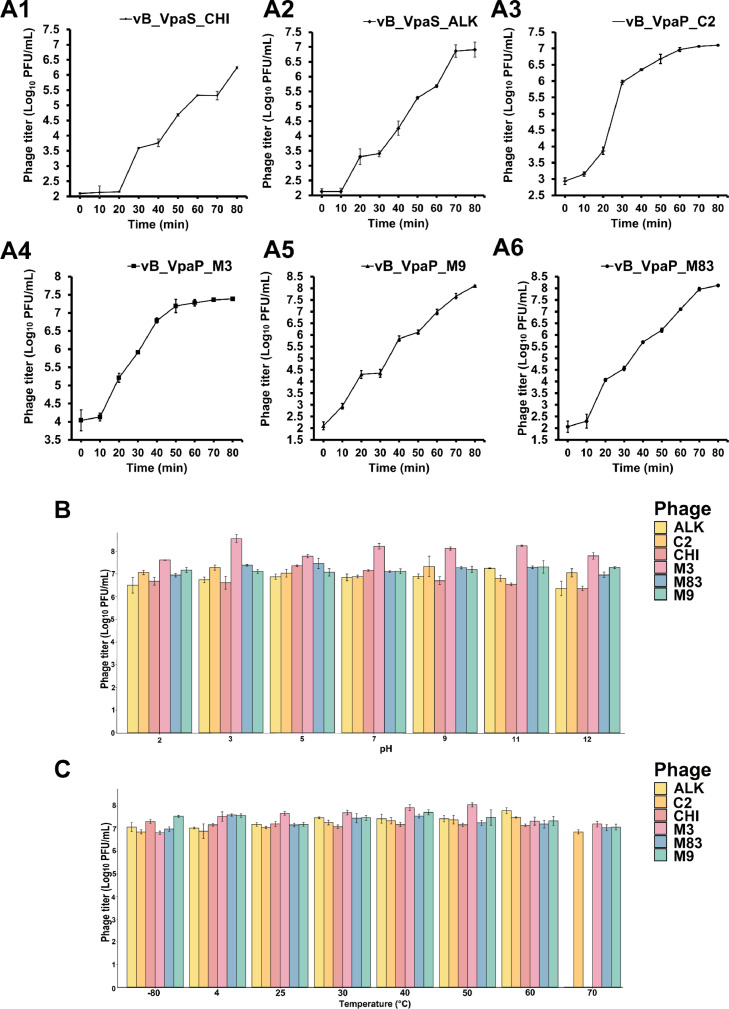
Biological characterization of phages. One-step growth curve of bacteriophages: (A1) CHI; (A2) ALK; (A3) C2; (A4) M3; (A5) M9; (A6) M83. (B) Stability of phages at various temperatures. (C) Stability of phages at various pH values. Significant differences in stability assays are in Supplementary TableS1 and TableS2. Values are the means of three tests ± standard deviation.

From the one-step growth curve, bacteriophages C2 and CHI showed a latent period of 20 min, while bacteriophages ALK, M83, and M3 showed a faster latent period of 10 min. Furthermore, bacteriophage M9 was observed with a latent period of 30 min (Fig. 2A). The average burst sizes of bacteriophages CHI, ALK, M3, C2, M9, and M83 estimated from the one-step growth curve were 111, 168, 35, 37, 34, and 35 PFU per cell, respectively.

### 3.4 Determination of phage stabilities

Phages M3, C2, M9, and M83 remained stable at -80, 4, and 25 °C and showed lytic activity at temperatures as high as 70 °C. In contrast, the phages CHI and ALK were stable between -80, 25, 4, and 60 °C. After 1 h of incubation at 70 °C, their activity was completely lost. The results of the pH stability test showed that the infectivity of ALK, CHI, M3, and M9 remained in the pH range of 2–11, but it was no longer active under alkaline conditions at pH 12. In comparison, phages M83 and C2 remained stable in the pH range of 2 to 9 with stability decreasing when the pH is 9. The results show that all phages can be used in harsh environments under different temperatures and pH conditions (Fig. 2B and C).

### 3.5 Basic genome analysis

**Fig. 3:**
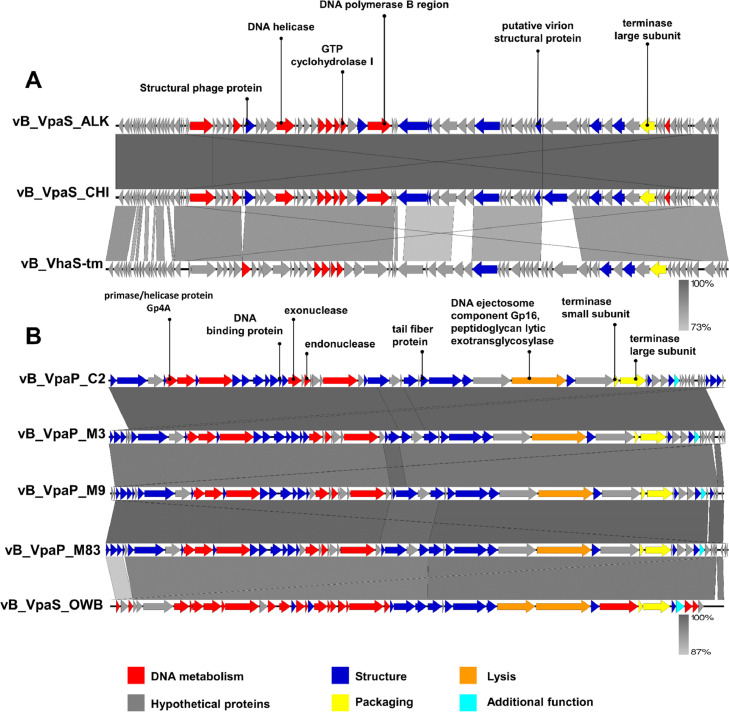
Genome comparison using Easyfig: (A) vB_VpaS_CHI, vB_VpaS_ALK, and vB_VhaS-tm. (B) vB_VpaP_M3, vB_VpaP_C2, vB_VpaP_M9, vB_VpaP_M83, and vB_VpaS_OWB. Different colored arrows represent predicted open reading frames with different functions.

All of the phages have a linear double-stranded DNA genome with lengths ranging between 43,268 and 57,805 bp. Their GC contents were 45.9% for ALK and CHI and 48.8% for M3, C2, M9, and M83 (Table 2). The CHI and ALK genomes contained 95 and 94 putative ORFs, with an average length of 215 bp and sizes ranging from 27 to 934 bp. In the same way, 57 and 58 putative ORFs, respectively, were predicted in the genomes of phages M3, M9, C2, and M83, with an average length of 273 bp and sizes ranging from 30 to 1284 bp. All predicted ORFs were divided into six modules (Fig. 3), including the DNA metabolism module, lysis module, packaging module, structure module, additional functions module, and the remaining allocated hypothetical proteins module. Briefly, to mention some of the predicted ORF functions, CHI ORF275 corresponds to a putative virion structural protein and showed 84% identity with a putative virion structural protein from phage pVco-14 (QQM14126.1). ORF9 and ORF128, both of the ALK phages, were identified as primase and tRNA ribosyltransferase, which showed a similarity of 94% with phage vB_VhaS-tm (ANO57534.1). ORF73 of phage M83 was identified as a phage protein that presented a similarity of 86% with phage KF1 (YP_009808040.1), and ORF21 of M9 corresponds to an endolysin, showing an identity of 98.99% with phage KF1. ORF24 of M3 was also identified as an endolysin that showed 99.5% similarity to phage OWB (YP_009948710.1). ORF26 corresponds to a hypothetical protein that showed a similarity of 94% with the phage MGD1 (QKK83123.1). The other main features of predicted ORFs for each phage are explained in detail in the Supplementary (Tables S3–S8). No tRNA genes were found using the tRNAscan-SE program. Compared with the ABRicate databases, no virulence or antibiotic resistance genes were found in the genomes of ALK, CHI, M3, C2, M9 or M83, indicating that they could be safely used to control *V. parahaemolyticus*, and the phage lifestyle was predicted to be virulent by PhageAI.

**Table 2:** Basic analysis of the phage genome.

Phage name	Genome length (bp)	GC content (%)	ORFs	tRNAs	Antibiotic resistance genes	Virulence genes
CHI	57,805	45.9	95	0	0	0
ALK	57,805	45.9	94	0	0	0
Tm	59,742	46.6	86	0	0	0
M3	43,446	48.8	58	0	0	0
M9	43,268	48.8	57	0	0	0
M83	43,901	48.8	57	0	0	0
C2	43,494	48.8	58	0	0	0
OWB	43,264	48.7	45	0	0	0

### 3.6 Phylogenetic and comparative genomic analysis

**Fig. 4:**
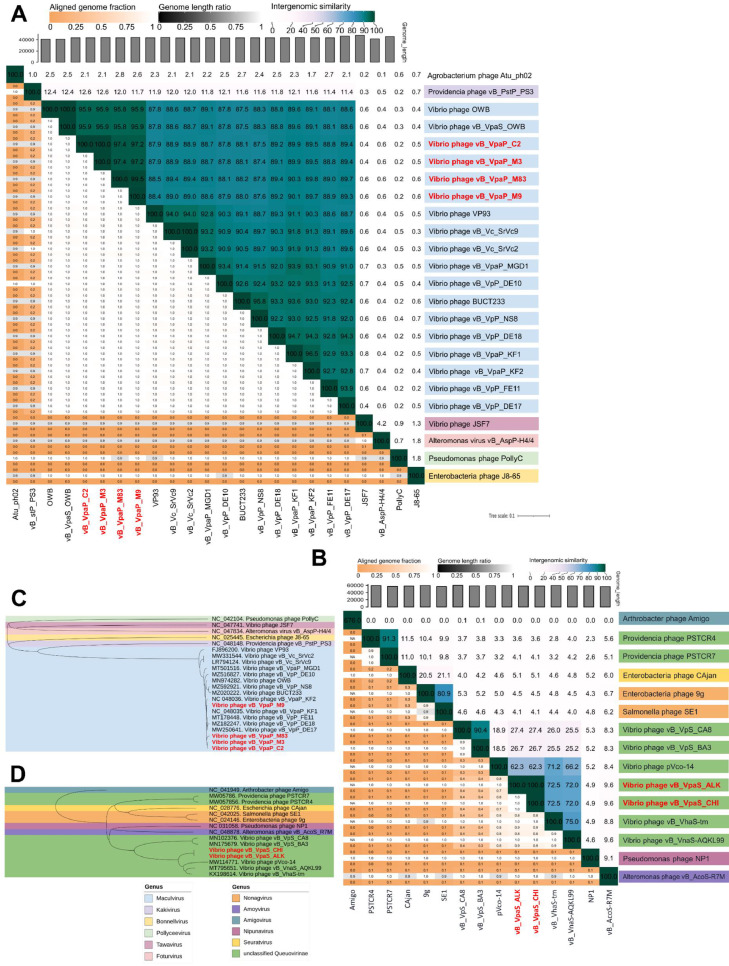
Heatmap of VIRIDIC: (A) M3, C2, M83, and M9. (B) CHI and ALK. The values of percentage identity range from 0 (0%, white) to 1 (100%, green). Phylogenetic tree based on Genome-BLAST Distance Phylogeny (GBDP) of (C) M3, C2, M83 and M9 of the *Autographiviridae* family. (D) CHI and ALK of the M3, C2, M83, and M9 of the *Queuovirina-e.* subfamily.

To investigate the evolutionary relationships between phages CHI and ALK and 15 other phages within the *Queuovirinae* subfamily, as well as between phages M3, C2, M9, and M83 and 24 other phages within the *Autographiviridae* family, two phylogenetic trees based on the whole genome were constructed using the Genome-BLAST Distance Phylogeny method (GBDP). Nucleotide sequence alignment based on blastn revealed that 2 phages had a nucleotide sequence coverage above 70% with CHI and ALK and identities below 95%, including vB_VnaS-AQKL99 (MT795651.1, 89.23% identity, and 77% coverage) and vB_VhaS-tm (KX198614.1, 86.56% identity, and 81% coverage). To further examine the relationship between CHI and ALK to 15 other long noncontractile tail vibriophages, intergenomic similarities among viral genomes were calculated through VIRIDIC with blastn defaults. As with the identity values, the ANI values between CHI and ALK to other phages were also below 95% (Fig. 4B). As shown in the phylogenetic tree (Fig. 4D), CHI and ALK were most closely related to the five phages, listed as vB_VnaS-AQKL99, vB_VhaS-tm, pVco-14, vB_VpS_CA8, and vB_VpS_BA3. These analyzes showed that phages CHI and ALK belong to a new genus not yet classified in the ICTV within the subfamily *Queuovirinae*, including these five phages that clustered on the same branch.

Alternatively, nucleotide sequence alignment based on blastn revealed that 14 phages had a nucleotide sequence coverage above 95% with vB_VpaP_M3, vB_VpaP_C2, vB_VpaP_M9 and vB_VpaP_M83, while identities were also above 90% (Supplementary Table S10), including OWB, vB_VpaS_OWB, vB_VpaP_KF1, vB_VpP_DE17, vB_VpP_DE18, vB_VpP_FE11, vB_Vc_SrVc2, vB_Vc_SrVc9, vB_VpP_DE10, vB_VpaP_MGD1, and vB_VpaP_KF2. To further examine the relationship between M3, C2, M9, and M83 and 24 other short tail vibriophages, intergenomic similarities among viral genomes were also calculated through the VIRIDIC with blastn defaults. Furthermore, as for the identity values, the ANI values between M3, C2, M9, and M83 to other phages were also above 95% (Fig. 4A). Moreover, as shown in the phylogenetic tree (Fig. 4C), M3, C2, M9, and M83 were most closely related to the thirteen phages previously listed. It is clear that M3, C2, M9, and M83 are closely related to these phages previously listed, and both belong to the genus *Maculvirus* in the *Autographiviridae* family. In summary, our study demonstrated that phages M3, C2, M9, and M83 were members of a new species in *Maculvirus*.

### 3.7 Bacterial challenge test

**Fig. 5:**
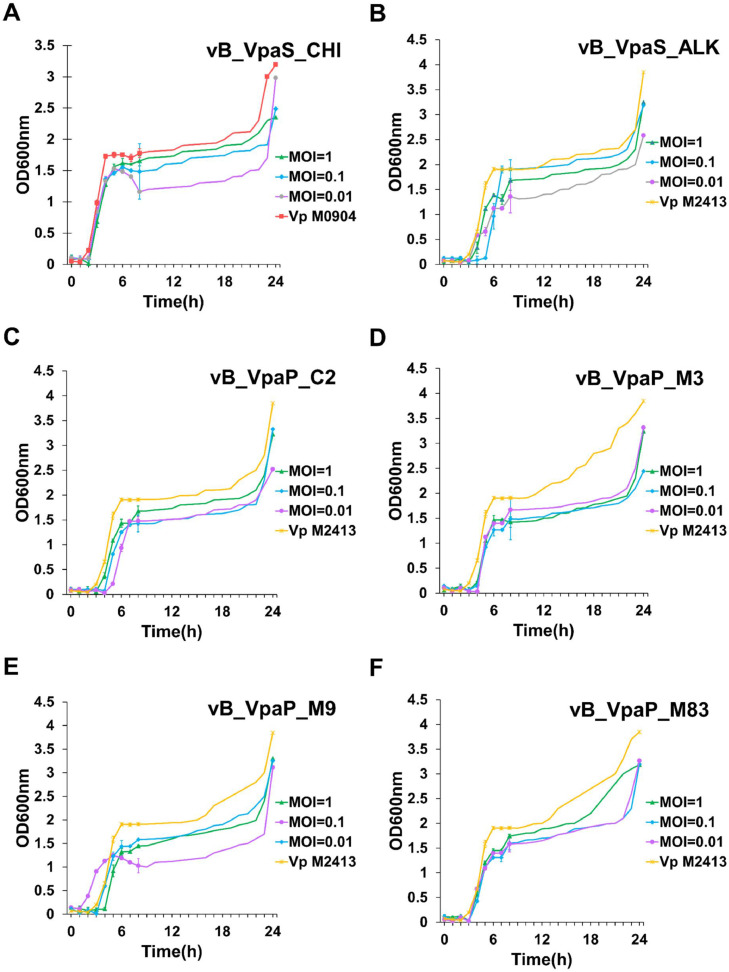
Bacterial challenge assay of phages: (A) CHI, (B) ALK, (C) C2, (D) M3, (E) M9, and (F) M83 to Mexican AHPND-causing *V. parahaemolyticus* strains according to different MOIs (MOI = 1, 0.1, 0.01).

*In vitro* antibacterial tests show that phages ALK, M3, C2, M9, and M83 can effectively inhibit the growth of strain M2413. Because of the bactericidal effect of phage CHI, it can effectively inhibit the growth of M0904. As shown in Fig. 5, ALK, M3, C2, M9, and M83 had some bactericidal effects after incubation for 3–8 h with MOIs of 1, 0.1, or 0.01. In contrast, CHI had a bactericidal effect within 2 h. Additionally, the higher the MOI is, the better the effect will be.

## 4 Discussion

Every available opportunity to counter the advance of antimicrobial resistance needs further exploration, and as Gordillo-Altamirano et al. (2019) noted, we are quickly approaching such a crisis: a so-called “postantibiotic era”. Therefore, in most cases, there are still effective compounds to eliminate bacterial infections. However, phage therapy must be ready as antimicrobial resistance becomes more pressing each day. In this sense, to achieve successful phage therapy, one needs to start with the isolation and characterization of numerous phages to have a deep understanding of the minimum suggested regulatory requirements of the phage in the study. To date, many studies have demonstrated the isolation and characterization of viable phages from environmental sources such as seawater, wastewater, and shellfish samples (Lomelí-Ortega and Martínez-Díaz, 2014; Jun et al., 2016; Yu et al., 2013; Wong et al., 2019; Matamp and Bhat, 2020). Even so, further work is needed to establish a large, specific, and reliable phage library that can provide phage specificity against the target pathogen. Additionally, we agree with Gordillo-Altamirano et al. (2019); we believe that the phage library can help to more quickly determine the identity of the bacterial host receptor of the phage, which will provide important information on the emergence of phage resistance, evolutionary trade-offs, and use of combination therapies that are less likely to generate phage-resistant hosts. In our study, six novel phages had lytic activity against Mexican AHPND-causing *V. parahaemolyticus* strains. The phages had some notable features, including short latency times and large burst sizes. According to Amarillas et al. (2017), a large burst size may represent a selective advantage as an antibacterial agent, because phages with a large burst size can increase the initial dose of phage several 100-fold in short periods. Cao et al. (2021) also reported that the burst size of MGD2 phage was 244 PFU/cell, which was considerably higher than that observed for most *V. parahaemolyticus* phages, including OMN, pVp-1, VP06, and VpKK5, producing approximately 18.67, 47, 60 and 180 PFU/cell, respectively. Compared to our vibriophages, the large burst size of these may be a definite advantage for their application in future *in vivo* experiments.

As another prerequisite for their use as biological control agents, phage survival conditions at different pH values and temperatures play an important role in phage attachment, penetration, and reproduction (Hu et al., 2021). CHI, ALK, M3, C2, M9, and M83 phages showed relatively broad pH and thermal stability compared to other vibriophages, such as PG07 (<60 °C, pH 3-11) (Ding et al., 2020). Therefore, there is a possibility that these six phages can be kept at room temperature and in frozen conditions. Furthermore, they exhibited higher tolerance to extreme pH values (Rong et al., 2014), suggesting a possible application in a harsh environment.

To better understand the general characteristics of the genome of these phages, we agree with Yang et al. (2020). The blastn analysis of our phages also showed that the genome sequence differences between these six phages and other phages with which we compared them were more than 5%. Therefore, we confirmed that our phages have a high similarity with the sequence of the other phages reported in the NCBI databases. Additionally, the genus demarcation of these six phages coincides in that the length of their genomes is established with a nucleotide identity greater than 70% (Turner et al., 2021), and in their case, the demarcation of species is also established with a nucleotide identity greater than 95% throughout the length of the genomes (Adriaenssens and Brister, 2017). The intergenomic similarities calculated between the viral genomes through the VIRIDIC platform with blastn defaults helped to support the relationship of the phages isolated in Sinaloa, Mexico, with the other phages with which the respective comparisons were established and properly recognize their taxonomic classification.

Phylogenetic analysis, intergenomic similarity heatmaps from VIRIDIC and phylogenetic trees revealed that the CHI and ALK phages are new members of a new genus not yet classified in the ICTV, within the subfamily *Queuovirinae*, class *Caudoviricetes*. The phages M3, C2, M9, and M83 are new phages of a new species not yet reported within the genus *Maculvirus*, family *Autographiviridae*. González-Gómez et al. (2022) reported that only two studies have described phages belonging to a new genus within the *Queuovirinae* subfamily (Hu et al., 2021; Yang et al., 2020). After the recent abolition of the order *Caudovirales* and the families *Myoviridae, Siphoviridae* and *Podoviridae*, we consider it necessary to prepare and present the proposal for a new genus and new species to the ICTV so that the new members of the genus and species can be further isolated and their genomes reported accordingly.

No tRNA-encoding genes were found in the genomes of these six phages, indicating that these phages depend on host tRNA for their protein synthesis (Ding et al., 2020; Cao et al., 2021). Although in our study, no antibiotic resistance, virulence, or lysogenic genes were found in any of the genomes, we agree with AL-Ishaq et al. (2021); more clinical studies and regulatory processes are required to evaluate its safety and efficacy as a biological control in future phage therapy.

Our results showed that CHI, ALK, M3, C2, M9, and M83 could infect 5 of 10 Mexican AHPND-causing *V. parahaemolyticus* strains, which was a relatively wide range. Notably, the *V. parahaemolyticus* isolates used in this study have not been assigned to any clonal complex but were previously subtyped through a robust phylogenomic analysis that revealed the evolutionary distance across these and other worldwide isolated strains of AHPND-*V. parahaemolyticus* (González Gómez et al., 2020). More robust studies such as the one by (Ding et al., 2020), report an infection rate of phage PG07 of 14 out of 30 AHPND-*V. parahaemolyticus* strains. González-Gómez et al. (2022), reported an infection rate of phage AL-1 of 2 of 10 and phage AL-2 4 of 10, both of Mexican AHPND-*V. parahaemolyticus* strains. However, we agree with Yang et al. (2022) that it was difficult to compare the host range of each phage because more strains than had been typed before were used in other studies, and the total number and range of hosts are different. However, we are the first to isolate and characterize novel vibriophages against AHPND-*V. parahaemolyticus* strains, due to the difficult task of isolating phages with lytic activity against AHPND-*V. parahaemolyticus*. Future studies might be representative trials including non-AHPND and AHPND-*V. parahaemolyticus* strains, given the low number of the last ones.

We confirmed the antimicrobial activity against the target pathogen. *In vitro* antibacterial tests showed that these six phages can effectively inhibit the growth of Mexican strains M0904 and M2413. However, we considered that these tests can still be improved because despite obtaining positive results *in vitro*, determining the kinetics of phages in animal models is necessary to identify key parameters of phages to design adequate prevention strategies for their use in phage therapy (Chang et al., 2018). The OD_600nm_ values of the culture at different MOIs gradually increased during the first hours and then decreased markedly compared to the respective positive control. We can predict that the bacteriolytic activity of these phages increased with increasing MOI, as observed in previous studies (Yang et al., 2020; Cao et al., 2021; Hu et al., 2021), but we also noticed a better effect the lower the MOI value (0.01, 0.1 and 1). In this case, the multiplicity of infection values are lower than those commonly reported from 1 to 100 (Cao et al., 2021). Ding et al. (2020) agree that the growth observed in the curves, during the first hours of incubation may be associated with the fact that bacteria could resist phage infection through various mechanisms, including spontaneous mutation, the restriction-modification system, and adaptive immunity through the CRISPR‒Cas system. In summary, conducting further model-based studies that include both *in vivo* and *in vitro* experiments is necessary to report the key parameters indicating that they are effective for use in therapy. The identity of the bacterial host receptor for any therapeutic phage should be established, and we must use combination therapies, such as phage-antibiotics, that are likely to generate antimicrobial resensitization. The obtained results show that phages CHI, ALK, M3, C2, M9, and M83 are suitable and promising alternatives to therapeutic agents to treat AHPND-*V. parahaemolyticus* strains.

## 5 Conclusion

Based on the basic physiological features analyzed, lytic activity and *in vitro* antimicrobial activity, these novel virulent vibriophages are suitable and promising candidates as biocontrol agents for Mexican AHPND-causing *V. parahaemolyticus* strains in future *in vivo* experiments. However, the development of phage therapy to treat AHPND in shrimp requires more studies. First, identifying the receptor is desirable, mainly due to the difficulty of isolating vibriophages against AHPND in Mexico. The second is the understanding of the dynamics of phage-bacterial-shrimp interactions in the *in vivo* context. Therefore, future studies could focus on the development of quantitative models or models of combination therapy coupling *in vitro* and *in vivo* experiments to characterize the interplay between phage and bacteria during AHPND to evaluate phage therapeutic efficacy in shrimp ponds.

## GenBank Accession Numbers

The complete genome sequences of vB_VpaS_CHI, vB_VpaS_ALK, vB_VpaP_M3, vB_VpaP_C2, vB_VpaP_M9, and vB_VpaP_M83 have been deposited in the GenBank database under the accession numbers ON457559, ON457558, ON457557, ON457556, ON457555, and ON457554, respectively.

## Funding

This work was supported by the Consejo Nacional de Ciencia y Tecnología (CONACyT) of Mexico through a scholarship granted to Alma Karen Orozco-Ochoa [No. 1083340].

## CRediT authorship contribution statement

**Alma Karen Orozco-Ochoa:** Conceptualization, Formal analysis, Methodology, Writing – original draft. **Jean Pierre González-Gómez:** Formal analysis, Methodology, Writing – review & editing. **Nohelia Castro-del Campo:** Conceptualization, Validation, Writing – review & editing. **Juan Daniel Lira-Morales:** Conceptualization, Validation, Writing – review & editing. **Célida Isabel Martínez-Rodríguez:** Conceptualization, Funding acquisition, Validation, Writing – review & editing. **Bruno Gomez-Gil:** Conceptualization, Funding acquisition, Validation, Writing – review & editing. **Cristóbal Chaidez:** Conceptualization, Funding acquisition, Validation, Writing – review & editing.

## Declaration of Competing Interest

The authors declare that they have no known competing financial interests or personal relationships that could have appeared to influence the work reported in this paper.

## Data Availability

Data will be made available on request.

## Appendix Supplementary materials

Image, application 1
