# Supplementary material for: Characterization and genome analysis of six novel Vibrio parahaemolyticus phages associated with acute hepatopancreatic necrosis disease (AHPND)
Source: Virus Res. 2022 Oct 20;323:198973. doi: 10.1016/j.virusres.2022.198973 (PMC10194199; doi:10.1016/j.virusres.2022.198973)
Supplement: Supplementary file 1 [file mmc1.docx]

| **Table S1. Grouping Information Using the Tukey Method and 95% Confidence for Temperature factor** | | | | |
| --- | --- | --- | --- | --- |
| **Phage strain**  **ID** | **Temperature** | **N** | **Mean** | **Grouping** |
| CHI | -80 | 3 | 7.3169 | A |
| CHI | 4 | 3 | 7.1720 | AB |
| CHI | 25 | 3 | 7.2036 | AB |
| CHI | 30 | 3 | 7.0867 | B |
| CHI | 40 | 3 | 7.1795 | AB |
| CHI | 50 | 3 | 7.1718 | AB |
| CHI | 60 | 3 | 7.1440 | AB |
| CHI | 70 | 3 | 0.0000 | C |
|  | | | | |
| ALK | -80 | 3 | 7.069 | c |
| ALK | 4 | 3 | 7.0281 | c |
| ALK | 25 | 3 | 7.1841 | bc |
| ALK | 30 | 3 | 7.4760 | ab |
| ALK | 40 | 3 | 7.437 | b |
| ALK | 50 | 3 | 7.4381 | b |
| ALK | 60 | 3 | 7.7894 | a |
| ALK | 70 | 3 | 0.0000 | d |
|  | | | | |
| M3 | -80 | 3 | 6.8198 | J |
| M3 | 4 | 3 | 7.535 | GHI |
| M3 | 25 | 3 | 7.6761 | FGH |
| M3 | 30 | 3 | 7.7103 | EFG |
| M3 | 40 | 3 | 7.9267 | EF |
| M3 | 50 | 3 | 8.0536 | E |
| M3 | 60 | 3 | 7.333 | HI |
| M3 | 70 | 3 | 7.2018 | I |
|  | | | | |
| C2 | -80 | 3 | 6.8592 | f |
| C2 | 4 | 3 | 6.884 | f |
| C2 | 25 | 3 | 7.0518 | ef |
| C2 | 30 | 3 | 7.2703 | ef |
| C2 | 40 | 3 | 7.3460 | e |
| C2 | 50 | 3 | 7.395 | e |
| C2 | 60 | 3 | 7.4908 | e |
| C2 | 70 | 3 | 6.8592 | f |
|  | | | | |
| M9 | -80 | 3 | 7.5462 | KL |
| M9 | 4 | 3 | 7.5790 | KL |
| M9 | 25 | 3 | 7.1903 | LM |
| M9 | 30 | 3 | 7.4814 | KLM |
| M9 | 40 | 3 | 7.7195 | K |
| M9 | 50 | 3 | 7.491 | KLM |
| M9 | 60 | 3 | 7.343 | KLM |
| M9 | 70 | 3 | 7.0666 | M |
|  | | |  | |
| M83 | -80 | 3 | 6.9792 | m |
| M83 | 4 | 3 | 7.6034 | k |
| M83 | 25 | 3 | 7.1625 | lm |
| M83 | 30 | 3 | 7.461 | kl |
| M83 | 40 | 3 | 7.5547 | k |
| M83 | 50 | 3 | 7.2535 | klm |
| M83 | 60 | 3 | 7.1992 | lm |
| M83 | 70 | 3 | 7.0428 | m |
| *Means that do not share a letter are significantly different.* | | | | |

| **Table S2. Grouping Information Using the Tukey Method and 95% Confidence for pH factor** | | | | |
| --- | --- | --- | --- | --- |
| **Phage strain**  **ID** | **pH** | **N** | **Mean** | **Grouping** |
| CHI | 2 | 3 | 6.678 | B |
| CHI | 3 | 3 | 6.615 | B |
| CHI | 5 | 3 | 7.3542 | A |
| CHI | 7 | 3 | 7.1554 | A |
| CHI | 9 | 3 | 6.702 | B |
| CHI | 11 | 3 | 6.5415 | B |
| CHI | 12 | 3 | 6.3597 | B |
|  | | | | |
| ALK | 2 | 3 | 6.502 | b |
| ALK | 3 | 3 | 6.7418 | ab |
| ALK | 5 | 3 | 6.8744 | ab |
| ALK | 7 | 3 | 6.8480 | ab |
| ALK | 9 | 3 | 6.8955 | ab |
| ALK | 11 | 3 | 7.2470 | a |
| ALK | 12 | 3 | 6.360 | b |
|  | | | | |
| M3 | 2 | 3 | 7.60912 | D |
| M3 | 3 | 3 | 8.553 | C |
| M3 | 5 | 3 | 7.7813 | E |
| M3 | 7 | 3 | 8.2149 | D |
| M3 | 9 | 3 | 8.1202 | D |
| M3 | 11 | 3 | 8.2422 | D |
| M3 | 12 | 3 | 7.7929 | E |
|  | | | | |
| C2 | 2 | 3 | 7.0634 | c |
| C2 | 3 | 3 | 7.2764 | c |
| C2 | 5 | 3 | 7.0335 | c |
| C2 | 7 | 3 | 6.8815 | c |
| C2 | 9 | 3 | 7.327 | c |
| C2 | 11 | 3 | 6.8105 | c |
| C2 | 12 | 3 | 7.053 | c |
|  | | | | |
| M9 | 2 | 3 | 7.1669 | G |
| M9 | 3 | 3 | 7.1082 | G |
| M9 | 5 | 3 | 7.0709 | G |
| M9 | 7 | 3 | 7.1167 | G |
| M9 | 9 | 3 | 7.1992 | G |
| M9 | 11 | 3 | 7.305 | G |
| M9 | 12 | 3 | 7.2771 | G |
|  | | |  | |
| M83 | 2 | 3 | 6.9492 | i |
| M83 | 3 | 3 | 7.3851 | gh |
| M83 | 5 | 3 | 7.458 | g |
| M83 | 7 | 3 | 7.1015 | hi |
| M83 | 9 | 3 | 7.2693 | gh |
| M83 | 11 | 3 | 7.2913 | gh |
| M83 | 12 | 3 | 6.9595 | i |
| *Means that do not share a letter are significantly different.* | | | | |

| **Table S3. Functional classification of 95 ORFs in the vB_VpaS_CHI genome** | | | | | | | | | | |
| --- | --- | --- | --- | --- | --- | --- | --- | --- | --- | --- |
| **Label** | **Strand** | **Start** | **Stop** | **Length nt\|aa** | **Function protein** | **Organism** | **Accession** | **Query Cover** | **E value** | **Identidy (%)** |
| vB_VpaS_CHI ORF155 | - | 29816 | 27012 | 2805 \| 934 | hypothetical protein | Phytophthora cactorum | KAF1795296.1 | 68% | 18 | 76.47% |
| vB_VpaS_CHI ORF154 | - | 30203 | 29988 | 216 \| 71 | hypothetical protein | Paenibacillus flagellatus | WP_146250266.1 | 82% | 0.73 | 58.62% |
| vB_VpaS_CHI ORF153 | - | 32666 | 31020 | 1647 \| 548 | hypothetical protein | Vibrio phage vB_VhaS-tm | ANO57534.1 | 100% | 0 | 97.81% |
| vB_VpaS_CHI ORF152 | - | 34343 | 33528 | 816 \| 271 | hypothetical protein | Edhazardia aedis USNM 41457 | EJW04853.1 | 34% | 56 | 90.00% |
| vB_VpaS_CHI ORF252 | - | 5640 | 5128 | 513 \| 170 | hypothetical protein | - | - | - | - | - |
| vB_VpaS_CHI ORF150 | - | 38552 | 38373 | 180 \| 59 | hypothetical protein | Perca fluviatilis | KAF1373361.1 | 62% | 7.6 | 65.00% |
| vB_VpaS_CHI ORF7 | + | 14317 | 15366 | 1050 \| 349 | hypothetical protein | Morchella conica CCBAS932 | RPB11477.1 | 60% | 0.039 | 63.16% |
| vB_VpaS_CHI ORF8 | + | 15379 | 17100 | 1722 \| 573 | primase | - | - | - | - | - |
| vB_VpaS_CHI ORF9 | + | 7150 | 9525 | 2376 \| 791 | hypothetical protein | Vibrio phage vB_VhaS-tm | ANO57534.1 | 100% | 0 | 95.07% |
| vB_VpaS_CHI ORF10 | + | 21526 | 22095 | 570 \| 189 | hypothetical protein | Vibrio phage vB_VhaS-tm | ANO57534.1 | 100% | 4.00E-180 | 98.78% |
| vB_VpaS_CHI ORF11 | + | 10741 | 11181 | 441 \| 146 | hypothetical protein | Vibrio phage PH669 | QQK88584.1 | 46% | 2.00E-51 | 62.59% |
| vB_VpaS_CHI ORF12 | + | 22501 | 22677 | 177 \| 58 | hypothetical protein | Vibrio phage vB_VnaS-AQKL99 | QMV29716.1 | 98% | 2.00E-100 | 86.16% |
| vB_VpaS_CHI ORF13 | + | 22858 | 23046 | 189 \| 62 | hypothetical protein | - | - | - | - | - |
| vB_VpaS_CHI ORF14 | + | 24088 | 26349 | 2262 \| 753 | Phage protein (ACLAME 618) | Vibrio phage vB_VnaS-AQKL99 | QMV29716.1 | 100% | 8.00E-127 | 92.42% |
| vB_VpaS_CHI ORF15 | + | 14317 | 15366 | 1050 \| 349 | hypothetical protein |  |  |  |  |  |
| vB_VpaS_CHI ORF18 | + | 33388 | 33576 | 189 \| 62 | hypothetical protein | Vibrio phage vB_VhaS-tm | ANO57534.1 | 100% | 0 | 94.50% |
| vB_VpaS_CHI ORF19 | + | 42439 | 42618 | 180 \| 59 | hypothetical protein |  |  |  |  |  |
| vB_VpaS_CHI ORF21 | + | 49786 | 50025 | 240 \| 79 | DNA helicase, phage associated; Type III restriction enzme | - | - | - | - | - |
| vB_VpaS_CHI ORF23 | + | 54628 | 54792 | 165 \| 54 | hypothetical protein | Vibrio phage vB_VnaS-AQKL99 | QMV29716.1 | 100% | 7.00E-91 | 93.06% |
| vB_VpaS_CHI ORF24 | + | 24088 | 26349 | 2262 \| 753 | hypothetical protein | Vibrio phage vB_VnaS-AQKL99 | QMV29716.1 | 84% | 2.00E-91 | 94.34% |
| vB_VpaS_CHI ORF25 | + | 55597 | 55761 | 165 \| 54 | hypothetical protein | Vibrio phage vB_VhaS-tm | ANO57534.1 | 46% | 8.00E-50 | 92.31% |
| vB_VpaS_CHI ORF27 | + | 57061 | 57282 | 222 \| 73 | hypothetical protein | Vibrio phage vB_VhaS-tm | ANO57534.1 | 95% | 7.00E-31 | 94.83% |
| vB_VpaS_CHI ORF28 | + | 29206 | 29322 | 117 \| 38 | 7-carboxy-7-deazaguanine synthase (EC 4.3.99.3) | - | - | - | - | - |
| vB_VpaS_CHI ORF29 | + | 1058 | 1297 | 240 \| 79 | 7-cyano-7-deazaguanine synthase (EC 6.3.4.20) | - | - | - | - | - |
| vB_VpaS_CHI ORF31 | + | 31531 | 31608 | 78 \| 25 | 6-carboxy-5,6,7,8-tetrahydropterin synthase (EC 4.1.2.50) | Vibrio phage vB_VhaS-tm | ANO57534.1 | 76% | 3.00E-47 | 91.67% |
| vB_VpaS_CHI ORF19 | + | 42439 | 42618 | 180 \| 59 | GTP cyclohydrolase I (EC 3.5.4.16) type 1 | - | - | - | - | - |
| vB_VpaS_CHI ORF32 | + | 32353 | 32619 | 267 \| 88 | hypothetical protein | - | - | - | - | - |
| vB_VpaS_CHI ORF34 | + | 33388 | 33576 | 189 \| 62 | hypothetical protein | Vibrio phage vB_VhaS-tm | ANO57534.1 | 87% | 4.00E-71 | 86.07% |
| vB_VpaS_CHI ORF38 | + | 28780 | 28953 | 174 \| 57 | Phage protein | Vibrio phage vB_VpS_BA3 | QEQ95113.1 | 100% | 5.00E-161 | 78.28% |
| vB_VpaS_CHI ORF39 | + | 29080 | 29121 | 42 \| 13 | tRNA ribosyltransferase [Vibrio phage vB_VnaS-AQKL99] | Vibrio phage vB_VnaS-AQKL99 | QMV29716.1 | 100% | 3.00E-58 | 53.11% |
| vB_VpaS_CHI ORF41 | + | 29461 | 29493 | 33 \| 10 | hypothetical protein | Vibrio phage pVco-14 | QQM14126.1 | 70% | 1.00E-22 | 69.86% |
| vB_VpaS_CHI ORF42 | + | 30778 | 30843 | 66 \| 21 | hypothetical protein | Caldilinea aerophila DSM 145 | BAL99815.1 | 52% | 27 | 90.91% |
| vB_VpaS_CHI ORF134 | + | 13835 | 14320 | 486 \| 161 | tail assembly protein | Staphylotrichum | KAG7290765.1 | 83% | 5.8 | 59.26% |
| vB_VpaS_CHI ORF132 | - | 4230 | 3910 | 321 \| 106 | tail assembly protein | Vibrio phage vB_VhaS-tm | ANO57534.1 | 100% | 0 | 94.50% |
| vB_VpaS_CHI ORF130 | - | 5640 | 5128 | 513 \| 170 | Phage protein | - | - | - | - | - |
| vB_VpaS_CHI ORF129 | - | 12201 | 12043 | 159 \| 52 | hypothetical protein | - | - | - | - | - |
| vB_VpaS_CHI ORF128 | - | 11403 | 11245 | 159 \| 52 | hypothetical protein | - | - | - | - | - |
| vB_VpaS_CHI ORF281 | - | 34343 | 33528 | 816 \| 271 | hypothetical protein | Vibrio phage vB_VnaS-AQKL99 | QMV29716.1 | 99% | 3.00E-33 | 33.45% |
| vB_VpaS_CHI ORF175 | - | 36643 | 34343 | 2301 \| 766 | tail length tape-measure protein 1 | Vibrio phage vB_VhaS-tm | ANO57534.1 | 100% | 0 | 86.81% |
| vB_VpaS_CHI ORF280 | - | 37058 | 36756 | 303 \| 100 | hypothetical protein | - | - | - | - | - |
| vB_VpaS_CHI ORF173 | - | 37441 | 37082 | 360 \| 119 | hypothetical protein | Halobacteriovoraceae bacterium | MAX66020.1 | 90% | 25 | 88.89% |
| vB_VpaS_CHI ORF172 | - | 37891 | 37454 | 438 \| 145 | hypothetical protein | - | - | - | - | - |
| vB_VpaS_CHI ORF225 | - | 38970 | 38038 | 933 \| 310 | hypothetical protein | Vibrio phage pVco-14 | QQM14126.1 | 100% | 0 | 90.65% |
| vB_VpaS_CHI ORF224 | - | 39399 | 38983 | 417 \| 138 | hypothetical protein | - | - | - | - | - |
| vB_VpaS_CHI ORF276 | - | 39800 | 39396 | 405 \| 134 | hypothetical protein | - | - | - | - | - |
| vB_VpaS_CHI ORF170 | - | 40219 | 39797 | 423 \| 140 | hypothetical protein | - | - | - | - | - |
| vB_VpaS_CHI ORF275 | - | 40697 | 40167 | 531 \| 176 | putative virion structural protein [Vibrio phage pVco-14] | Vibrio phage pVco-14 | QQM14126.1 | 100% | 2.00E-108 | 84.09% |
| vB_VpaS_CHI ORF167 | - | 40927 | 40754 | 174 \| 57 | hypothetical protein | - | - | - | - | - |
| vB_VpaS_CHI ORF223 | - | 43287 | 41014 | 2274 \| 757 | hypothetical protein | Vibrio phage SHOU24 | YP_009006419.1 | 99% | 0 | 49.04% |
| vB_VpaS_CHI ORF222 | - | 44328 | 43291 | 1038 \| 345 | hypothetical protein | Vibrio phage pVco-14 | QQM14126.1 | 97% | 4.00E-135 | 60.18% |
| vB_VpaS_CHI ORF272 | - | 45209 | 44628 | 582 \| 193 | hypothetical protein | Trichodelitschia | KAF2402602.1 | 83% | 64 | 52.00% |
| vB_VpaS_CHI ORF140 | + | 32024 | 32059 | 36 \| 11 | hypothetical protein | Anaerolineaceae bacterium | NMC85007.1 | 90% | 31 | 76.92% |
| vB_VpaS_CHI ORF220 | - | 45801 | 45493 | 309 \| 102 | Phage major capsid protein of Caudovirales | Salvia splendens | KAG6396615.1 | 100% | 9.2 | 90.00% |
| vB_VpaS_CHI ORF217 | - | 47619 | 46738 | 882 \| 293 | hypothetical protein | Vibrio phage vB_VhaS-tm | ANO57534.1 | 100% | 0 | 93.17% |
| vB_VpaS_CHI ORF266 | - | 48794 | 48642 | 153 \| 50 | minor head protein-like protein | - | - | - | - | - |
| vB_VpaS_CHI ORF265 | - | 50231 | 48807 | 1425 \| 474 | hypothetical protein | Vibrio phage vB_VhaS-tm | ANO57534.1 | 100% | 0 | 91.98% |
| vB_VpaS_CHI ORF160 | - | 51709 | 50243 | 1467 \| 488 | terminase large subunit | Vibrio phage vB_VhaS-tm | ANO57534.1 | 100% | 0 | 97.75% |
| vB_VpaS_CHI ORF211 | - | 52350 | 51931 | 420 \| 139 | hypothetical protein | Vibrio phage vB_VhaS-tm | ANO57534.1 | 46% | 8.00E-50 | 92.31% |
| vB_VpaS_CHI ORF43 | + | 52516 | 52614 | 99 \| 32 | hypothetical protein | Vibrio phage pVco-14 | QQM14126.1 | 100% | 1.00E-69 | 80.58% |
| vB_VpaS_CHI ORF263 | - | 53141 | 52647 | 495 \| 164 | N-acetylmuramoyl-L-alanine amidase | Vibrio phage vB_VhaS-tm | ANO57534.1 | 99% | 4.00E-104 | 86.50% |
| vB_VpaS_CHI ORF158 | - | 53692 | 53147 | 546 \| 181 | hypothetical protein | Vibrio phage vB_VhaS-tm | ANO57534.1 | 100% | 6.00E-126 | 95.03% |
| vB_VpaS_CHI ORF157 | - | 54058 | 53807 | 252 \| 83 | hypothetical protein | Vibrio phage vB_VhaS-tm | ANO57534.1 | 89% | 5.00E-39 | 82.43% |
| vB_VpaS_CHI ORF206 | - | 54294 | 54028 | 267 \| 88 | hypothetical protein | Vibrio phage pVco-14 | QQM14126.1 | 98% | 1.00E-36 | 70.11% |
| vB_VpaS_CHI ORF261 | - | 54902 | 54306 | 597 \| 198 | hypothetical protein | - | - | - | - | - |
| vB_VpaS_CHI ORF149 | + | 54963 | 55127 | 165 \| 54 | hypothetical protein | - | - | - | - | - |
| vB_VpaS_CHI ORF153 | - | 32666 | 31020 | 1647 \| 548 | hypothetical protein | Vibrio phage vB_VhaS-tm | ANO57534.1 | 100% | 0 | 97.81% |
| vB_VpaS_CHI ORF260 | - | 56987 | 56529 | 459 \| 152 | hypothetical protein | Vibrio phage vB_VhaS-tm | ANO57534.1 | 100% | 2.00E-83 | 90.79% |
| vB_VpaS_CHI ORF203 | - | 57228 | 57043 | 186 \| 61 | hypothetical protein | Vibrio phage vB_VhaS-tm | ANO57534.1 | 100% | 6.00E-34 | 90.16% |
| vB_VpaS_CHI ORF259 | - | 57635 | 57306 | 330 \| 109 | hypothetical protein | Vibrio phage vB_VnaS-AQKL99 | QMV29716.1 | 100% | 3.00E-70 | 97.25% |

| **Table S4. Functional classification of 94 ORFs in the vB_VpaS_ALK genome** | | | | | | | | | | |
| --- | --- | --- | --- | --- | --- | --- | --- | --- | --- | --- |
| **Label** | **Strand** | **Start** | **Stop** | **Length nt\|aa** | **Function protein** | **Organism** | **Accession** | **Query Cover** | **E value** | **Identidy (%)** |
| vB_VpaS_ALK ORF 260 | + | 44013 | 44057 | 45 \| 14 | hypothetical protein | *Vibrio* phage vB_VhaS-tm | ANO57534.1 | 100% | 9.00E-61 | 81.13% |
| vB_VpaS_ALK ORF309 | - | 40030 | 39995 | 36 \| 11 | hypothetical protein | *Bulinus truncatus* | KAH9514266.1 | 90% | 30 | 90.00% |
| vB_VpaS_ALK ORF259 | + | 43857 | 43895 | 39 \| 12 | hypothetical protein | *Planctomycetes bacterium* | MBS0202587.1 | 100% | 13 | 83.33% |
| vB_VpaS_ALK ORF199 | + | 3663 | 3764 | 102 \| 33 | hypothetical protein | - | - | - | - | - |
| vB_VpaS_ALK ORF258 | + | 43779 | 43832 | 54 \| 17 | hypothetical protein | *Anaerolineaceae bacterium* | MBI1280530.1 | 82% | 5.7 | 71.43% |
| vB_VpaS_ALK ORF198 | + | 3339 | 3455 | 117 \| 38 | hypothetical protein | - | - | - | - | - |
| vB_VpaS_ALK ORF60 | + | 6875 | 7147 | 273 \| 90 | hypothetical protein | - | - | - | - | - |
| vB_VpaS_ALK ORF9 | + | 7150 | 9363 | 2214 \| 737 | primase | *Vibrio* phage vB_VhaS-tm | ANO57534.1 | 100% | 0 | 94.72% |
| vB_VpaS_ALK ORF113 | + | 17405 | 17974 | 570 \| 189 | hypothetical protein | *Vibrio* phage vB_VnaS-AQKL99 | QMV29716.1 | 84% | 2.00E-91 | 94.34% |
| vB_VpaS_ALK ORF114 | - | 48807 | 47644 | 1164 \| 387 | hypothetical protein | - | - | - | - | - |
| vB_VpaS_ALK ORF62 | + | 49786 | 50025 | 240 \| 79 | hypothetical protein | - | - | - | - | - |
| vB_VpaS_ALK ORF115 | - | 47619 | 46738 | 882 \| 293 | hypothetical protein | *Vibrio* phage vB_VhaS-tm | ANO57534.1 | 46% | 8.00E-50 | 92.31% |
| vB_VpaS_ALK ORF13 | + | 7150 | 9363 | 2214 \| 737 | ATPase | *Vibrio* phage vB_VhaS-tm | ANO57534.1 | 100% | 0 | 94.72% |
| vB_VpaS_ALK ORF116 | - | 46329 | 46177 | 153 \| 50 | hypothetical protein | *Chloroflexi bacterium* | MCE9646809.1 | 78% | 1.2 | 90.91% |
| vB_VpaS_ALK ORF16 | + | 10027 | 10110 | 84 \| 27 | Phage protein (ACLAME 618) | *Lactarius sangui* | KAH9164212.1 | 59% | 2.9 | 58.33% |
| vB_VpaS_ALK ORF118 | - | 45426 | 45226 | 201 \| 66 | hypothetical protein | *Catenaria anguillulae* PL171 | ORZ39287.1 | 86% | 78 | 65.00% |
| vB_VpaS_ALK ORF119 | + | 14394 | 15443 | 1050 \| 349 | hypothetical protein | *Vibrio* phage vB_VnaS-AQKL99 | QMV29716.1 | 100% | 0 | 94.27% |
| vB_VpaS_ALK ORF120 | + | 15456 | 17177 | 1722 \| 573 | hypothetical protein | *Vibrio* phage vB_VhaS-tm | ANO57534.1 | 100% | 0 | 95.29% |
| vB_VpaS_ALK ORF23 | + | 53188 | 53364 | 177 \| 58 | DNA helicase, phage associated; Type III restriction enzme | *Vibrio* phage vB_VnaS-AQKL99 | QMV29716.1 | 98% | 2.00E-100 | 86.16% |
| vB_VpaS_ALK ORF121 | + | 21521 | 21559 | 39 \| 12 | hypothetical protein | - | - | - | - | - |
| vB_VpaS_ALK ORF24 | + | 14779 | 14871 | 93 \| 30 | hypothetical protein | *Brevundimonas* sp. | HCW49610.1 | 91% | 4.6 | 90.91% |
| vB_VpaS_ALK ORF68 | + | 22578 | 22754 | 177 \| 58 | hypothetical protein | *Sphingomonas* | MBB4881513.1 | 60% | 2.9 | 73.68% |
| vB_VpaS_ALK ORF25 | + | 55330 | 55482 | 153 \| 50 | hypothetical protein | - | - | - | - | - |
| vB_VpaS_ALK ORF69 | + | 22935 | 23123 | 189 \| 62 | 7-carboxy-7-deazaguanine synthase (EC 4.3.99.3) | *Vibrio* phage vB_VnaS-AQKL99 | QMV29716.1 | 95% | 0 | 95.97% |
| vB_VpaS_ALK ORF122 | - | 40296 | 39874 | 423 \| 140 | 7-cyano-7-deazaguanine synthase (EC 6.3.4.20) | *Vibrio* phage vB_VhaS-tm | ANO57534.1 | 95% | 7.00E-31 | 94.83% |
| vB_VpaS_ALK ORF247 | - | 21162 | 21043 | 120 \| 39 | 6-carboxy-5,6,7,8-tetrahydropterin synthase (EC 4.1.2.50) | - | - | - | - | - |
| vB_VpaS_ALK ORF123 | + | 21480 | 21602 | 123 \| 40 | GTP cyclohydrolase I (EC 3.5.4.16) type 1 | - | - | - | - | - |
| vB_VpaS_ALK ORF124 | - | 37968 | 37531 | 438 \| 145 | hypothetical protein | *Vibrio* phage pVco-14 | QQM14126.1 | 100% | 2.00E-128 | 92.06% |
| vB_VpaS_ALK ORF70 | + | 24165 | 26426 | 2262 \| 753 | hypothetical protein | *Vibrio* phage vB_VhaS-tm | ANO57534.1 | 100% | 0 | 94.23% |
| vB_VpaS_ALK ORF28 | + | 23185 | 24168 | 984 \| 327 | Phage protein | *Vibrio* phage vB_VhaS-tm | ANO57534.1 | 100% | 0 | 94.50% |
| vB_VpaS_ALK ORF128 | + | 26741 | 26860 | 120 \| 39 | tRNA ribosyltransferase [Vibrio phage vB_VnaS-AQKL99] | *Vibrio* phage vB_VhaS-tm | ANO57534.1 | 100% | 0 | 94.42% |
| vB_VpaS_ALK ORF129 | - | 28341 | 28138 | 204 \| 67 | hypothetical protein | *Vibrio* phage VP06 | AVI05139.1 | 100% | 9.00E-34 | 32.04% |
| vB_VpaS_ALK ORF74 | + | 33465 | 33653 | 189 \| 62 | hypothetical protein | *Vibrio* phage vB_VhaS-tm | ANO57534.1 | 95% | 6.00E-25 | 62.20% |
| vB_VpaS_ALK ORF178 | - | 29893 | 27089 | 2805 \| 934 | tail assembly protein | *Vibrio* phage vB_VnaS-AQKL99 | QMV29716.1 | 85% | 0 | 80.25% |
| vB_VpaS_ALK ORF78 | + | 54639 | 54872 | 234 \| 77 | tail assembly protein | - | - | - | - | - |
| vB_VpaS_ALK ORF177 | - | 30280 | 30065 | 216 \| 71 | Phage protein | *Vibrio* phage vB_VnaS-AQKL99 | QMV29716.1 | 100% | 2.00E-34 | 94.37% |
| vB_VpaS_ALK ORF238 | - | 31095 | 30292 | 804 \| 267 | hypothetical protein | *Vibrio* phage vB_VhaS-tm | ANO57534.1 | 100% | 1.00E-34 | 85.71% |
| vB_VpaS_ALK ORF287 | - | 33605 | 32736 | 870 \| 289 | hypothetical protein | - | - | - | - | - |
| vB_VpaS_ALK ORF174 | - | 34420 | 33605 | 816 \| 271 | hypothetical protein | *Thaumasiovibrio occultus* | WP_086937497.1 | 99% | 3.00E-38 | 34.77% |
| vB_VpaS_ALK ORF81 | - | 54580 | 54398 | 183 \| 60 | tail length tape-measure protein 1 | - | - | - | - | - |
| vB_VpaS_ALK ORF173 | - | 37135 | 36833 | 303 \| 100 | hypothetical protein | *Vibrio* phage pVco-14 | QQM14126.1 | 100% | 2.00E-58 | 87.00% |
| vB_VpaS_ALK ORF273 | - | 48197 | 48114 | 84 \| 27 | hypothetical protein | *Desulfobacula* sp. | MCF6248740.1 | 44% | 2.20E+00 | 78.57% |
| vB_VpaS_ALK ORF232 | - | 37968 | 37531 | 438 \| 145 | hypothetical protein | *Vibrio* phage pVco-14 | QQM14126.1 | 97% | 6.00E-48 | 61.27% |
| vB_VpaS_ALK ORF282 | + | 56847 | 56912 | 66 \| 21 | hypothetical protein | *Vibrio* phage pVco-14 | QQM14126.1 | 100% | 0 | 90.65% |
| vB_VpaS_ALK ORF281 | + | 56256 | 56387 | 132 \| 43 | hypothetical protein | *Vibrio* phage vB_VnaS-AQKL99 | QMV29716.1 | 100% | 2.00E-82 | 86.96% |
| vB_VpaS_ALK ORF169 | - | 39877 | 39473 | 261 \| 86 | hypothetical protein | *Vibrio* phage vB_VhaS-tm | ANO57534.1 | 100% | 4.00E-80 | 85.07% |
| vB_VpaS_ALK ORF230 |  | 40296 | 39874 | 423 \| 140 | hypothetical protein | *Vibrio* phage vB_VhaS-tm | ANO57534.1 | 87% | 4.00E-71 | 86.07% |
| vB_VpaS_ALK ORF168 | - | 40774 | 40244 | 531 \| 176 | putative virion structural protein | *Vibrio* phage pVco-14 | QQM14126.1 | 100% | 2.00E-108 | 84.09% |
| vB_VpaS_ALK ORF227 |  | 41004 | 40831 | 174 \| 57 | hypothetical protein | *Vibrio* phage pVco-14 | QQM14126.1 | 84% | 9.00E-19 | 79.17% |
| vB_VpaS_ALK ORF226 |  | 43287 | 41014 | 2274 \| 757 | hypothetical protein | *Vibrio* phage SHOU24 | YP_009006419.1 | 99% | 0 | 49.04% |
| vB_VpaS_ALK ORF225 | - | 44328 | 43291 | 1038 \| 345 | hypothetical protein | *Vibrio* phage vB_VnaS-AQKL99 | QMV29716.1 | 96% | 8.00E-138 | 60.60% |
| vB_VpaS_ALK ORF278 |  | 45209 | 44628 | 582 \| 193 | hypothetical protein | *Vibrio* phage vB_VhaS-tm | ANO57534.1 | 100% | 1.00E-82 | 73.10% |
| vB_VpaS_ALK ORF141 | - | 53141 | 52647 | 495 \| 164 | hypothetical protein | *Ignelater luminosus* | KAF2883916.1 | 100% | 61 | 69.23% |
| vB_VpaS_ALK ORF165 | - | 43505 | 43537 | 33 \| 10 | Phage major capsid protein of Caudovirales | *Kiritimatiellae bacterium* | MBQ6327425.1 | 80% | 143 | 100.00% |
| vB_VpaS_ALK ORF220 | + | 20799 | 20870 | 72 \| 23 | hypothetical protein | *Vibrio* phage vB_VhaS-tm | ANO57534.1 | 100% | 0 | 93.17% |
| vB_VpaS_ALK ORF219 | + | 20442 | 20516 | 75 \| 24 | minor head protein-like protein | *Neolentinus lepideus* HHB14362 ss-1 | KZT25546.1 | 91% | 0.38 | 51.52% |
| vB_VpaS_ALK ORF271 | - | 50231 | 48807 | 1425 \| 474 | hypothetical protein | *Vibrio* phage vB_VhaS-tm | ANO57534.1 | 100% | 0 | 91.98% |
| vB_VpaS_ALK ORF251 | + | 35799 | 35831 | 33 \| 10 | terminase large subunit | *Cyanobacteria bacterium* J055 | RMG05825.1 | 90% | 13 | 88.89% |
| vB_VpaS_ALK ORF270 | + | 53010 | 53087 | 78 \| 25 | hypothetical protein | *Hyalomma asiaticum* | KAH6926663.1 | 56% | 4.7 | 78.57% |
| vB_VpaS_ALK ORF214 | + | 13467 | 13952 | 486 \| 161 | hypothetical protein | *Vibrio* phage vB_VhaS-tm | ANO57534.1 | 100% | 2.00E-108 | 97.52% |
| vB_VpaS_ALK ORF160 | - | 41960 | 41998 | 39 \| 12 | hypothetical protein | *Massilia umbonata* | WP_137315416.1 | 83% | 106 | 90.91% |
| vB_VpaS_ALK ORF269 | + | 52392 | 52580 | 189 \| 62 | N-acetylmuramoyl-L-alanine amidase | - | - | - | - | - |
| vB_VpaS_ALK ORF159 | - | 12278 | 12120 | 159 \| 52 | hypothetical protein | - | - | - | - | - |
| vB_VpaS_ALK ORF158 | - | 15395 | 15006 | 390 \| 129 | hypothetical protein | - | - | - | - | - |
| vB_VpaS_ALK ORF209 | + | 4839 | 4573 | 267 \| 88 | hypothetical protein | *Vibrio* phage vB_VhaS-tm | ANO57534.1 | 100% | 5.00E-39 | 72.73% |
| vB_VpaS_ALK ORF267 | + | 50688 | 50819 | 132 \| 43 | hypothetical protein | - | - | - | - | - |
| vB_VpaS_ALK ORF150 | - | 35639 | 35298 | 342 \| 113 | hypothetical protein | - | - | - | - | - |
| vB_VpaS_ALK ORF154 | + | 37202 | 37429 | 228 \| 75 | hypothetical protein | - | - | - | - | - |
| vB_VpaS_ALK ORF266 | + | 50208 | 50246 | 39 \| 12 | hypothetical protein | *Vibrio* phage vB_VnaS-AQKL99 | QMV29716.1 | 100% | 2.00E-83 | 90.79% |
| vB_VpaS_ALK ORF206 | + | 8970 | 9074 | 105 \| 34 | hypothetical protein | - | - | - | - | - |
| vB_VpaS_ALK ORF265 | + | 47640 | 47678 | 39 \| 12 | hypothetical protein | *Vibrio* phage vB_VnaS-AQKL99 | QMV29716.1 | 100% | 3.00E-70 | 97.25% |

| **Table S5. Functional classification of 57 ORFs in the vB_VpaP_M83 genome** | | | | | | | | | | |
| --- | --- | --- | --- | --- | --- | --- | --- | --- | --- | --- |
| **Label** | **Strand** | **Start** | **Stop** | **Length nt\|aa** | **Function protein** | **Organism** | **Accession** | **Query Cover** | **E value** | **Identidy (%)** |
| vB_VpaP_M83 ORF73 | + | 30 | 293 | 264 \| 87 | Phage protein | *Vibrio* phage vB_VpaP_KF1 | YP_009808040.1 | 100% | 2.00E-49 | 86.21% |
| vB_VpaP_M83 ORF1 | + | 343 | 876 | 534 \| 177 | Phage protein | *Vibrio* phage vB_VpaS_OWB | YP_009948710.1 | 100% | 5.00E-127 | 100.00% |
| vB_VpaP_M83 ORF74 | - | 30725 | 30231 | 495 \| 164 | Phage protein | *Cyanothece* sp. | NET30419.1 | 73% | 11 | 81.82% |
| vB_VpaP_M83 ORF36 | + | 1199 | 1348 | 150 \| 49 | Phage protein | *Mangifera indica* | XP_044511269.1 | 80% | 6.3 | 100.00% |
| vB_VpaP_M83 ORF75 | + | 1350 | 1607 | 258 \| 85 | hypothetical protein | *Vibrio* phage vB_VpaS_OWB | YP_009948710.1 | 100% | 2.00E-55 | 100.00% |
| vB_VpaP_M83 ORF35 | + | 809 | 1189 | 381 \| 126 | Phage protein | *Vibrio* phage vB_VpaS_OWB | YP_009948710.1 | 100% | 2.00E-134 | 100.00% |
| vB_VpaP_M83 ORF76 | + | 2040 | 4175 | 2136 \| 711 | Phage protein | - | - | - | - | - |
| vB_VpaP_M83 ORF8 | + | 4216 | 5319 | 1104 \| 367 | hypothetical protein | *Vibrio* phage vB_VpaS_OWB | YP_009948710.1 | 100% | 0 | 100.00% |
| vB_VpaP_M83 ORF9 | + | 5344 | 5508 | 165 \| 54 | Phage protein | *Vibrio* phage vB_Vc_SrVc2 | QQM14916.1 | 99% | 1.00E-135 | 96.89% |
| vB_VpaP_M83 ORF77 | + | 5505 | 6320 | 816 \| 271 | Phage primase/helicase protein Gp4A | - | - | - | - | - |
| vB_VpaP_M83 ORF41 | + | 6302 | 7582 | 1281 \| 426 | Phage DNA helicase | *Vibrio* phage vB_VpaP_KF1 | YP_009808040.1 | 100% | 8.00E-50 | 98.99% |
| vB_VpaP_M83 ORF43 | + | 7808 | 10234 | 2427 \| 808 | Phage protein | *Vibrio* phage vB_VpaS_OWB | YP_009948710.1 | 100% | 2.00E-81 | 98.54% |
| vB_VpaP_M83 ORF86 | + | 10170 | 10823 | 654 \| 217 | Phage DNA-directed DNA polymerase (EC 2.7.7.7 | - | - | - | - | - |
| vB_VpaP_M83 ORF44 | + | 10832 | 11428 | 597 \| 198 | Phage protein | *Vibrio* phage VP93 | YP_002875667.1 | 30% | 0.006 | 68.42% |
| vB_VpaP_M83 ORF45 | + | 11630 | 12442 | 813 \| 270 | Phage protein | *Vibrio* phage vB_VpaP_KF1 | YP_009808040.1 | 100% | 2.00E-49 | 86.21% |
| vB_VpaP_M83 ORF17 | + | 40285 | 40899 | 615 \| 204 | Phage protein p21 | *Vibrio* phage vB_VpaP_KF2 | YP_009808068.1 | 100% | 1.00E-140 | 98.04% |
| vB_VpaP_M83 ORF90 | + | 12831 | 13460 | 630 \| 209 | Phage protein | *Vibrio* phage vB_VpaS_OWB | YP_009948710.1 | 100% | 2.00E-55 | 100.00% |
| vB_VpaP_M83 ORF46 | + | 13460 | 13663 | 204 \| 67 | Phage DNA binding protein | - | - | - | - | - |
| vB_VpaP_M83 ORF47 | + | 13673 | 14098 | 426 \| 141 | Phage protein | *Vibrio* phage vB_VpaS_OWB | YP_009948710.1 | 100% | 0 | 96.77% |
| vB_VpaP_M83 ORF19 | + | 809 | 1189 | 381 \| 126 | hypothetical protein | *Vibrio* phage VP93 | YP_002875667.1 | 81% | 3.00E-66 | 95.15% |
| vB_VpaP_M83 ORF49 | + | 15038 | 15244 | 207 \| 68 | hypothetical protein | - | - | - | - | - |
| vB_VpaP_M83 ORF93 | + | 15231 | 15671 | 441 \| 146 | Phage exonuclease (EC 3.1.11.3) | - | - | - | - | - |
| vB_VpaP_M83 ORF50 | + | 15671 | 15790 | 120 \| 39 | hypothetical protein | - | - | - | - | - |
| vB_VpaP_M83 ORF20 | + | 15787 | 16371 | 585 \| 194 | hypothetical protein | - | - | - | - | - |
| vB_VpaP_M83 ORF94 | + | 16338 | 16544 | 207 \| 68 | hypothetical protein | - | - | - | - | - |
| vB_VpaP_M83 ORF95 | + | 16560 | 19010 | 2451 \| 816 | Phage DNA-directed RNA polymerase (EC 2.7.7.6) | - | - | - | - | - |
| vB_VpaP_M83 ORF24 | + | 19198 | 19461 | 264 \| 87 | hypothetical protein | *Vibrio* phage vB_Vc_SrVc2 | QQM14901.1 | 100% | 0 | 98.83% |
| vB_VpaP_M83 ORF52 | + | 19448 | 19693 | 246 \| 81 | Phage protein | *Vibrio* phage vB_VpaS_OWB | YP_009948710.1 | 100% | 8.00E-124 | 100.00% |
| vB_VpaP_M83 ORF25 | + | 19702 | 21234 | 1533 \| 510 | Phage collar, head-to-tail connector protein Gp8 | - | - | - | - | - |
| vB_VpaP_M83 ORF97 | + | 21234 | 22052 | 819 \| 272 | hypothetical protein | - | - | - | - | - |
| vB_VpaP_M83 ORF57 | + | 22118 | 22819 | 702 \| 233 | Phage major capsid protein Gp10A | *Vibrio* phage vB_VpP_DE17 | QPP19730.1 | 100% | 2.00E-157 | 93.38% |
| vB_VpaP_M83 ORF28 | + | 22789 | 23793 | 1005 \| 334 | Phage major capsid protein Gp10A | *Vibrio* phage vB_VpaS_OWB | YP_009948710.1 | 84% | 5.00E-159 | 99.56% |
| vB_VpaP_M83 ORF99 | - | 40002 | 39838 | 165 \| 54 | hypothetical protein | - | - | - | - | - |
| vB_VpaP_M83 ORF58 | + | 27465 | 27620 | 156 \| 51 | Phage tail fiber protein | - | - | - | - | - |
| vB_VpaP_M83 ORF59 | + | 24569 | 26911 | 2343 \| 780 | hypothetical protein | - | - | - | - | - |
| vB_VpaP_M83 ORF60 | + | 26921 | 27667 | 747 \| 248 | Phage protein | *Vibrio* phage vB_VpaS_OWB | YP_009948710.1 | 100% | 5.00E-145 | 98.52% |
| vB_VpaP_M83 ORF61 | + | 27677 | 30355 | 2679 \| 892 | hypothetical protein | - | - | - | - | - |
| vB_VpaP_M83 ORF29 | + | 30406 | 34260 | 3855 \| 1284 | Phage DNA ejectosome component Gp16, peptidoglycan lytic exotransglycosylase | *Vibrio* phage vB_VpaS_OWB | YP_009948710.1 | 100% | 6.00E-41 | 98.51% |
| vB_VpaP_M83 ORF107 | + | 34281 | 34892 | 612 \| 203 | Phage tail fiber protein | - | - | - | - | - |
| vB_VpaP_M83 ORF65 | + | 34901 | 37633 | 2733 \| 910 | hypothetical protein | *Vibrio* phage VP93 | YP_002875667.1 | 94% | 3.00E-22 | 72.06% |
| vB_VpaP_M83 ORF66 | - | 41879 | 41715 | 165 \| 54 | Phage terminase small subunit Gp18, DNA packaging | - | - | - | - | - |
| vB_VpaP_M83 ORF123 | - | 38177 | 37992 | 186 \| 61 | Phage terminase large subunit Gp19, DNA packaging" | - | - | - | - | - |
| vB_VpaP_M83 ORF68 | + | 39977 | 40276 | 300 \| 99 | hypothetical protein | - | - | - | - | - |
| vB_VpaP_M83 ORF32 | + | 40285 | 40899 | 615 \| 204 | Phage protein | *Vibrio* phage vB_VpaP_KF2 | YP_009808068.1 | 100% | 1.00E-140 | 98.04% |
| vB_VpaP_M83 ORF114 | + | 40899 | 41453 | 555 \| 184 | hypothetical protein | - | - | - | - | - |
| vB_VpaP_M83 ORF70 | + | 41462 | 41875 | 414 \| 137 | Phage protein | *Vibrio* phage vB_VpaS_OWB | YP_009948710.1 | 100% | 2.00E-81 | 98.54% |
| vB_VpaP_M83 ORF116 | - | 5982 | 5803 | 180 \| 59 | gene 66 protein | *Vibrio* phage vB_VpP_DE10 | QXV72189.1 | 100% | 3.00E-80 | 99.15% |
| vB_VpaP_M83 ORF117 | + | 42333 | 42551 | 219 \| 72 | hypothetical protein | *Vibrio* phage VP93 | YP_002875667.1 | 94% | 3.00E-22 | 72.06% |

| **Table S6. Functional classification of 57 ORFs in the vB_VpaP_M9 genome** | | | | | | | | | | |
| --- | --- | --- | --- | --- | --- | --- | --- | --- | --- | --- |
| **Label** | **Strand** | **Start** | **Stop** | **Length nt \| aa** | **Function protein** | **Organism** | **Accession** | **Query Cover** | **E value** | **Identidy (%)** |
| vB_VpaP_M9 ORF35 | + | 16949 | 19399 | 2451 \| 816 | hypothetical protein | *Vibrio* phage vB_VpaP_KF1 | YP_009808040.1 | 100% | 2.00E-49 | 86.21% |
| vB_VpaP_M9 ORF81 | - | 40570 | 40253 | 318 \| 105 | hypothetical protein | *Vibrio* phage vB_VpaS_OWB | YP_009948710.1 | 100% | 5.00E-127 |  |
| vB_VpaP_M9 ORF36 | + | 20168 | 21700 | 1533 \| 510 | hypothetical protein | *Vibrio* phage vB_VpaS_OWB | YP_009948710.1 | 100% | 0 | 99.41% |
| vB_VpaP_M9 ORF2 | + | 2206 | 2418 | 213 \| 70 | hypothetical protein | *Vibrio* phage VP93 | YP_002875667.1 | 100% | 3.00E-25 | 100.00% |
| vB_VpaP_M9 ORF37 | + | 22346 | 22591 | 246 \| 81 | hypothetical protein | - | - | - | - |  |
| vB_VpaP_M9 ORF83 | - | 37009 | 36755 | 255 \| 84 | hypothetical protein | - | - | - | - |  |
| vB_VpaP_M9 ORF38 | + | 27260 | 27415 | 156 \| 51 | hypothetical protein | - | - | - | - |  |
| vB_VpaP_M9 ORF88 | - | 30520 | 30026 | 495 \| 164 | hypothetical protein | - | - | - | - |  |
| vB_VpaP_M9 ORF89 | - | 24352 | 24185 | 168 \| 55 | hypothetical protein | - | - | - | - |  |
| vB_VpaP_M9 ORF39 | + | 28409 | 28564 | 156 \| 51 | hypothetical protein | - | - | - | - |  |
| vB_VpaP_M9 ORF7 | + | 8026 | 8193 | 168 \| 55 | hypothetical protein | - | - | - | - |  |
| vB_VpaP_M9 ORF9 | + | 11221 | 11817 | 597 \| 198 | hypothetical protein | *Vibrio* phage vB_VpaS_OWB | YP_009948710.1 | 100% | 6.00E-131 | 97.47% |
| vB_VpaP_M9 ORF48 | + | 4605 | 5708 | 1104 \| 367 | hypothetical protein | *Vibrio* phage vB_VpaS_OWB | YP_009948710.1 | 100% | 0 | 96.73% |
| vB_VpaP_M9 ORF10 | + | 12019 | 12831 | 813 \| 270 | hypothetical protein | *Vibrio* phage vB_VpaS_OWB | YP_009948710.1 | 84% | 5.00E-159 | 99.56% |
| vB_VpaP_M9 ORF11 | + | 13849 | 14052 | 204 \| 67 | hypothetical protein | *Vibrio* phage vB_VpaS_OWB | YP_009948710.1 | 100% | 6.00E-41 | 98.51% |
| vB_VpaP_M9 ORF97 | + | 12876 | 13208 | 333 \| 110 | hypothetical protein | - | - | - | - | - |
| vB_VpaP_M9 ORF52 | + | 14490 | 15440 | 951 \| 316 | hypothetical protein | *Vibrio* phage vB_VpaS_OWB | YP_009948710.1 | 100% | 0 | 100.00% |
| vB_VpaP_M9 ORF12 | + | 13849 | 14052 | 204 \| 67 | hypothetical protein | *Vibrio* phage VP93 | YP_002875667.1 | 100% | 2.00E-67 | 99.29% |
| vB_VpaP_M9 ORF13 | + | 14062 | 14487 | 426 \| 141 | hypothetical protein | *Vibrio* phage vB_VpaS_OWB | YP_009948710.1 | 100% | 4.00E-42 | 100.00% |
| vB_VpaP_M9 ORF99 | - | 39108 | 38830 | 279 \| 92 | hypothetical protein | - | - | - | - | - |
| vB_VpaP_M9 ORF15 | + | 23794 | 24354 | 561 \| 186 | hypothetical protein | *Vibrio* phage vB_VpaS_OWB | YP_009948710.1 | 100% | 2.00E-134 | 100.00% |
| vB_VpaP_M9 ORF55 | + | 19587 | 19769 | 183 \| 60 | hypothetical protein | *Vibrio* phage vB_VpP_DE17 | QPP19730.1 | 98% | 1.00E-34 | 100.00% |
| vB_VpaP_M9 ORF16 | + | 24364 | 26706 | 2343 \| 780 | holin | *Vibrio* phage vB_VpaS_OWB | YP_009948710.1 | 100% | 0 | 99.74% |
| vB_VpaP_M9 ORF100 | - | 36027 | 35806 | 222 \| 73 | hypothetical protein | - | - | - | - | - |
| vB_VpaP_M9 ORF56 | + | 19914 | 20159 | 246 \| 81 | hypothetical protein | *Vibrio* phage vB_VpaP_KF2 | YP_009808068.1 | 100% | 6.00E-33 | 83.82% |
| vB_VpaP_M9 ORF57 | + | 20634 | 20810 | 177 \| 58 | hypothetical protein | - | - | - | - | - |
| vB_VpaP_M9 ORF58 | + | 22584 | 23588 | 1005 \| 334 | hypothetical protein | *Vibrio* phage vB_VpP_FE11 | QIW87194.1 | 100% | 1.00E-19 | 100.00% |
| vB_VpaP_M9 ORF106 | + | 19914 | 20159 | 246 \| 81 | hypothetical protein | *Vibrio* phage vB_Vc_SrVc2 | QQM14929.1 | 100% | 0 | 99.22% |
| vB_VpaP_M9 ORF59 | + | 30201 | 34055 | 3855 \| 1284 | hypothetical protein | *Vibrio* phage vB_VpaS_OWB | YP_009948710.1 | 100% | 0 | 98.79% |
| vB_VpaP_M9 ORF19 | + | 34696 | 37428 | 2733 \| 910 | hypothetical protein | - | - | - | - | - |
| vB_VpaP_M9 ORF111 | - | 11232 | 10969 | 264 \| 87 | hypothetical protein | - | - | - | - | - |
| vB_VpaP_M9 ORF62 | + | 42294 | 42557 | 264 \| 87 | hypothetical protein | *Vibrio* phage vB_VpaS_OWB | YP_009948710.1 | 83% | 9.00E-49 | 100.00% |
| vB_VpaP_M9 ORF20 | + | 37438 | 37737 | 300 \| 99 | u-spanin | *Vibrio* phage vB_VpP_DE18 | QWY13587.1 | 86% | 5.00E-14 | 47.86% |
| vB_VpaP_M9 ORF21 | + | 39772 | 40071 | 300 \| 99 | endolysin | *Vibrio* phage vB_VpaP_KF1 | YP_009808040.1 | 100% | 8.00E-50 | 98.99% |
| vB_VpaP_M9 ORF22 | + | 40153 | 40380 | 228 \| 75 | holin | - | - | - | - | - |
| vB_VpaP_M9 ORF23 | + | 41257 | 41670 | 414 \| 137 | hypothetical protein | *Vibrio* phage vB_VpaS_OWB | YP_009948710.1 | 100% | 2.00E-81 | 98.54% |
| vB_VpaP_M9 ORF112 | - | 3540 | 3337 | 204 \| 67 | hypothetical protein | - | - | - | - | - |
| vB_VpaP_M9 ORF70 | - | 25520 | 25281 | 240 \| 79 | putative phosphoesterase | - | - | - | - | - |
| vB_VpaP_M9 ORF27 | + | 5894 | 6709 | 816 \| 271 | hypothetical protein | *Vibrio* phage vB_VpaS_OWB | YP_009948710.1 | 99% | 0 | 97.41% |
| vB_VpaP_M9 ORF28 | + | 37438 | 37737 | 300 \| 99 | hypothetical protein | - | - | - | - | - |
| vB_VpaP_M9 ORF76 | - | 9752 | 9492 | 261 \| 86 | hypothetical protein | - | - | - | - | - |
| vB_VpaP_M9 ORF30 | + | 10559 | 11212 | 654 \| 217 | hypothetical protein | *Vibrio* phage vB_Vc_SrVc2 | QQM14929.1 | 88% | 1.00E-137 | 97.38% |
| vB_VpaP_M9 ORF115 | + | 40080 | 40694 | 615 \| 204 | hypothetical protein | *Vibrio* phage vB_VpaP_KF2 | YP_009808068.1 | 100% | 1.00E-140 | 98.04% |
| vB_VpaP_M9 ORF77 | - | 9452 | 9189 | 264 \| 87 | hypothetical protein | - | - | - | - | - |
| vB_VpaP_M9 ORF32 | + | 14090 | 14251 | 162 \| 53 | DNA helicase | - | - | - | - | - |
| vB_VpaP_M9 ORF69 | - | 28382 | 28164 | 219 \| 72 | hypothetical protein | - | - | - | - | - |

| **Table S7. Functional classification of 58 ORFs in the vB_VpaP_M3 genome** | | | | | | | | | | |
| --- | --- | --- | --- | --- | --- | --- | --- | --- | --- | --- |
| **Label** | **Strand** | **Start** | **Stop** | **Length nt\|aa** | **Function protein** | **Organism** | **Accession** | **Query Cover** | **E value** | **Identidy (%)** |
| vB_VpaP_M3 ORF38 | + | 74 | 337 | 264 \| 87 | hypothetical protein | *Vibrio* phage vB_VpaS_OWB | YP_009948710.1 | 100% | 0 | 99.88% |
| vB_VpaP_M3 ORF1 | + | 373 | 906 | 534 \| 177 | hypothetical protein | *Vibrio* phage vB_VpaS_OWB | YP_009948710.1 | 100% | 5.00E-127 | 100.00% |
| vB_VpaP_M3 ORF90 | + | 918 | 1022 | 105 \| 34 | hypothetical protein | - | - | - | - | - |
| vB_VpaP_M3 ORF2 | + | 1228 | 1365 | 138 \| 45 | hypothetical protein | - | - | - | - | - |
| vB_VpaP_M3 ORF41 | + | 1367 | 1624 | 258 \| 85 | hypothetical protein | - | - | - | - | - |
| vB_VpaP_M3 ORF92 | + | 1596 | 2027 | 432 \| 143 | hypothetical protein | - | - | - | - | - |
| vB_VpaP_M3 ORF42 | + | 2057 | 4195 | 2139 \| 712 | hypothetical protein | - | - | - | - | - |
| vB_VpaP_M3 ORF99 | + | 4236 | 5339 | 1104 \| 367 | hypothetical protein | - | - | - | - | - |
| vB_VpaP_M3 ORF100 | + | 5364 | 5528 | 165 \| 54 | hypothetical protein | - | - | - | - | - |
| vB_VpaP_M3 ORF43 | + | 5525 | 6340 | 816 \| 271 | hypothetical protein | - | - | - | - | - |
| vB_VpaP_M3 ORF7 | + | 6322 | 7602 | 1281 \| 426 | hypothetical protein | - | - | - | - | - |
| vB_VpaP_M3 ORF103 | + | 7602 | 7835 | 234 \| 77 | hypothetical protein | - | - | - | - | - |
| vB_VpaP_M3 ORF268 | - | 8455 | 8291 | 165 \| 54 | hypothetical protein | - | - | - | - | - |
| vB_VpaP_M3 ORF52 | + | 10190 | 10843 | 654 \| 217 | hypothetical protein | *Vibrio* phage vB_VpaS_OWB | YP_009948710.1 | 84% | 9.00E-158 | 98.69% |
| vB_VpaP_M3 ORF10 | + | 10852 | 11448 | 597 \| 198 | hypothetical protein | *Vibrio* phage vB_VpaS_OWB | YP_009948710.1 | 100% | 3.00E-89 | 100.00% |
| vB_VpaP_M3 ORF11 | + | 11650 | 12462 | 813 \| 270 | hypothetical protein | *Vibrio* phage vB_VpaP_KF1 | YP_009808040.1 | 100% | 2.00E-42 | 97.10% |
| vB_VpaP_M3 ORF108 | + | 12507 | 12839 | 333 \| 110 | hypothetical protein | - | - | - | - | - |
| vB_VpaP_M3 ORF55 | + | 12851 | 13480 | 630 \| 209 | hypothetical protein | *Vibrio* phage vB_VpP_FE11 | QIW87194.1 | 100% | 3.00E-31 | 100.00% |
| vB_VpaP_M3 ORF12 | + | 13480 | 13689 | 210 \| 69 | hypothetical protein | *Vibrio* phage vB_VpaP_MGD1 | QKK83123.1 | 100% | 5.00E-91 | 94.33% |
| vB_VpaP_M3 ORF13 | + | 13699 | 14124 | 426 \| 141 | hypothetical protein | *Vibrio* phage vB_VpaS_OWB | YP_009948710.1 | 100% | 4.00E-42 | 100.00% |
| vB_VpaP_M3 ORF109 | + | 14127 | 15077 | 951 \| 316 | hypothetical protein | - | - | - | - | - |
| vB_VpaP_M3 ORF15 | + | 15064 | 15270 | 207 \| 68 | hypothetical protein | - | - | - | - | - |
| vB_VpaP_M3 ORF59 | + | 15257 | 15697 | 441 \| 146 | hypothetical protein | *Vi*brio phage vB_VpaS_OWB | YP_009948710.1 | 99% | 3.00E-137 | 98.45% |
| vB_VpaP_M3 ORF16 | + | 15697 | 15816 | 120 \| 39 | holin | *Vibrio* phage vB_VpP_DE17 | QPP19730.1 | 100% | 8.00E-159 | 94.12% |
| vB_VpaP_M3 ORF110 | + | 15813 | 16397 | 585 \| 194 | hypothetical protein | - | - | - | - | - |
| vB_VpaP_M3 ORF60 | + | 33397 | 33447 | 51 \| 16 | hypothetical protein | - | - | - | - | - |
| vB_VpaP_M3 ORF61 | + | 16586 | 19036 | 2451 \| 816 | hypothetical protein | *Vibrio* phage vB_VpP_DE17 | QPP19730.1 | 100% | 2.00E-57 | 100.00% |
| vB_VpaP_M3 ORF201 | - | 19748 | 19326 | 423 \| 140 | hypothetical protein | *Vibrio* phage vB_VpaP_KF1 | YP_009808040.1 | 35% | 1.00E-11 | 93.88% |
| vB_VpaP_M3 ORF18 | + | 19474 | 19719 | 246 \| 81 | hypothetical protein | *Vibrio* phage vB_VpaP_KF1 | YP_009808040.1 | 100% | 1.00E-48 | 100.00% |
| vB_VpaP_M3 ORF114 | + | 19728 | 20537 | 810 \| 269 | hypothetical protein | *Vibrio* phage vB_VpaS_OWB | YP_009948710.1 | 89% | 9.00E-164 | 99.58% |
| vB_VpaP_M3 ORF275 | - | 4414 | 3695 | 720 \| 239 | hypothetical protein | - | - | - | - | - |
| vB_VpaP_M3 ORF22 | + | 21337 | 22155 | 819 \| 272 | hypothetical protein | *Vibrio* phage vB_VpP_DE17 | QPP19730.1 | 100% | 8.00E-159 | 94.12% |
| vB_VpaP_M3 ORF117 | + | 22221 | 23219 | 999 \| 332 | hypothetical protein | *Vibrio* phage vB_VpP_DE17 | QPP19730.1 | 100% | 0 | 98.80% |
| vB_VpaP_M3 ORF66 | + | 23231 | 23359 | 129 \| 42 | hypothetical protein | *Vibrio* phage vB_VpaS_OWB | YP_009948710.1 | 100% | 1.00E-19 | 97.62% |
| vB_VpaP_M3 ORF23 | + | 23425 | 23985 | 561 \| 186 | u-spanin | *Vibrio* phage vB_VpaS_OWB | YP_009948710.1 | 100% | 2.00E-134 | 100.00% |
| vB_VpaP_M3 ORF24 | + | 23995 | 26337 | 2343 \| 780 | endolysin | *Vibrio* phage vB_VpaS_OWB | YP_009948710.1 | 100% | 0 | 99.49% |
| vB_VpaP_M3 ORF257 | - | 20752 | 20606 | 147 \| 48 | holin | - | - | - | - | - |
| vB_VpaP_M3 ORF26 | + | 27103 | 29781 | 2679 \| 892 | hypothetical protein | *Vibrio* phage vB_VpaS_OWB | YP_009948710.1 | 100% | 0 | 99.78% |
| vB_VpaP_M3 ORF118 | + | 29832 | 33686 | 3855 \| 1284 | hypothetical protein | *Vibrio* phage vB_VpaS_OWB | YP_009948710.1 | 100% | 0 | 99.53% |
| vB_VpaP_M3 ORF80 | + | 33707 | 34318 | 612 \| 203 | putative phosphoesterase | *Vibrio* phage vB_VpaS_OWB | YP_009948710.1 | 100% | 8.00E-146 | 99.01% |
| vB_VpaP_M3 ORF30 | + | 34327 | 37059 | 2733 \| 910 | hypothetical protein | *Vibrio* phage vB_VpaS_OWB | YP_009948710.1 | 100% | 0 | 98.68% |
| vB_VpaP_M3 ORF31 | + | 37069 | 37368 | 300 \| 99 | hypothetical protein | *Vibrio* phage vB_VpaS_OWB | YP_009948710.1 | 83% | 9.00E-49 | 100.00% |
| vB_VpaP_M3 ORF34 | + | 39784 | 40011 | 228 \| 75 | hypothetical protein | - | - | - | - | - |
| vB_VpaP_M3 ORF33 | + | 39403 | 39702 | 300 \| 99 | hypothetical protein | *Vibrio* phage vB_VpaP_MGD1 | QKK83123.1 | 100% | 1.00E-49 | 98.99% |
| vB_VpaP_M3 ORF120 | + | 39711 | 40325 | 615 \| 204 | hypothetical protein | *Vibrio* phage vB_VpaS_OWB | YP_009948710.1 | 100% | 8.00E-141 | 98.53% |
| vB_VpaP_M3 ORF85 | + | 40325 | 40879 | 555 \| 184 | hypothetical protein | *Vibrio* phage vB_VpaS_OWB | YP_009948710.1 | 100% | 5.00E-129 | 96.74% |
| vB_VpaP_M3 ORF35 | + | 40888 | 41301 | 414 \| 137 | DNA helicase | *Vibrio* phage vB_VpaS_OWB | YP_009948710.1 | 100% | 2.00E-81 | 98.54% |
| vB_VpaP_M3 ORF87 | + | 41294 | 41650 | 357 \| 118 | hypothetical protein | *Vibrio* phage vB_VpP_DE10 | QXV72189.1 | 100% | 3.00E-80 | 99.15% |

| **Table S8. Functional classification of 58 ORFs in the vB_VpaP_C2 genome** | | | | | | | | | | |
| --- | --- | --- | --- | --- | --- | --- | --- | --- | --- | --- |
| **Label** | **Strand** | **Start** | **Stop** | **Length nt\|aa** | **Function protein** | **Organism** | **Accession** | **Query Cover** | **E value** | **Identidy (%)** |
| vB_VpaP_C2 ORF1 | + | 145 | 576 | 432 \| 143 | hypothetical protein | *Vibrio* phage vB_VpaS_OWB | YP_009948710.1 | 100% | 3.00E-89 | 100.00% |
| vB_VpaP_C2 ORF3 | + | 1693 | 1788 | 96 \| 31 | hypothetical protein | *Vibrio* phage vB_VpaS_OWB | YP_009948710.1 | 100% | 0 | 96.73% |
| vB_VpaP_C2 ORF5 | + | 2407 | 2523 | 117 \| 38 | hypothetical protein | *Vibrio* phage vB_VpaS_OWB | YP_009948710.1 | 100% | 9.00E-51 | 100.00% |
| vB_VpaP_C2 ORF8 | + | 2785 | 3888 | 1104 \| 367 | hypothetical protein | *Vibrio* phage vB_VpaS_OWB | YP_009948710.1 | 99% | 3.00E-137 | 98.45% |
| vB_VpaP_C2 ORF9 | + | 3913 | 4077 | 165 \| 54 | hypothetical protein | - | - | - | - | - |
| vB_VpaP_C2 ORF11 | + | 4720 | 4815 | 96 \| 31 | hypothetical protein | *Vibrio* phage vB_VpaS_OWB | YP_009948710.1 | 100% | 0 | 99.41% |
| vB_VpaP_C2 ORF13 | + | 6931 | 7011 | 81 \| 26 | hypothetical protein | *Vibrio* phage vB_VpP_DE17 | QPP19730.1 | 100% | 0 | 98.80% |
| vB_VpaP_C2 ORF15 | + | 9199 | 9303 | 105 \| 34 | hypothetical protein | *Vibrio* phage vB_VpaS_OWB | YP_009948710.1 | 100% | 8.00E-141 | 98.53% |
| vB_VpaP_C2 ORF17 | + | 11056 | 11388 | 333 \| 110 | hypothetical protein | - | - | - | - | - |
| vB_VpaP_C2 ORF18 | + | 12676 | 13626 | 951 \| 316 | hypothetical protein | - | - | - | - | - |
| vB_VpaP_C2 ORF19 | + | 14362 | 14946 | 585 \| 194 | hypothetical protein | *Vibrio* phage VP93 | YP_002875667.1 | 100% | 1.00E-19 | 84.75% |
| vB_VpaP_C2 ORF20 | + | 16138 | 16359 | 222 \| 73 | hypothetical protein | *Vibrio* phage vB_VpP_DE18 | QWY13549.1 | 100% | 2.00E-57 | 83.96% |
| vB_VpaP_C2 ORF21 | + | 17398 | 17493 | 96 \| 31 | hypothetical protein | *Vibrio* phage vB_Vc_SrVc2 | QQM14901.1 | 100% | 0 | 98.83% |
| vB_VpaP_C2 ORF22 | + | 17773 | 18036 | 264 \| 87 | hypothetical protein | - | - | - | - | - |
| vB_VpaP_C2 ORF23 | + | 18277 | 19809 | 1533 \| 510 | hypothetical protein | *Vibrio* phage vB_VpaS_OWB | YP_009948710.1 | 100% | 0 | 98.51% |
| vB_VpaP_C2 ORF25 | + | 20455 | 20700 | 246 \| 81 | hypothetical protein | *Vibrio* phage vB_VpaS_OWB | YP_009948710.1 | 84% | 9.00E-158 | 98.69% |
| vB_VpaP_C2 ORF26 | + | 20818 | 21816 | 999 \| 332 | hypothetical protein | *Vibrio* phage vB_VpaP_MGD1 | QKK83123.1 | 100% | 5.00E-91 | 94.33% |
| vB_VpaP_C2 ORF27 | + | 28429 | 32283 | 3855 \| 1284 | hypothetical protein | *Vibrio* phage vB_Vc_SrVc2 | QQM14901.1 | 88% | 1.00E-137 | 97.38% |
| vB_VpaP_C2 ORF45 | + | 14246 | 14365 | 120 \| 39 | holin | *Vibrio* phage vB_VpaS_OWB | YP_009948710.1 | 100% | 4.00E-42 | 100.00% |
| vB_VpaP_C2 ORF28 | + | 13613 | 13819 | 207 \| 68 | hypothetical protein | *Vibrio* phage vB_VpaS_OWB | YP_009948710.1 | 100% | 4.00E-42 | 100.00% |
| vB_VpaP_C2 ORF29 | + | 38308 | 38922 | 615 \| 204 | hypothetical protein | *Vibrio* phage vB_VpaP_KF1 | YP_009808040.1 | 100% | 1.00E-48 | 100.00% |
| vB_VpaP_C2 ORF30 | + | 40420 | 40662 | 243 \| 80 | hypothetical protein | - | - | - | - | - |
| vB_VpaP_C2 ORF32 | + | 383 | 595 | 213 \| 70 | hypothetical protein | *Vibrio* phage vB_VpaS_OWB | YP_009948710.1 | 100% | 0 | 99.49% |
| vB_VpaP_C2 ORF47 | + | 18023 | 18268 | 246 \| 81 | hypothetical protein | - | - | - | - | - |
| vB_VpaP_C2 ORF34 | + | 3680 | 3859 | 180 \| 59 | hypothetical protein | *Vibrio* phage vB_VpaS_OWB | YP_009948710.1 | 100% | 0 | 99.78% |
| vB_VpaP_C2 ORF36 | + | 4871 | 6151 | 1281 \| 426 | hypothetical protein | *Vibrio* phage vB_VpaS_OWB | YP_009948710.1 | 83% | 9.00E-49 | 100.00% |
| vB_VpaP_C2 ORF38 | + | 6377 | 8803 | 2427 \| 808 | hypothetical protein | - | - | - | - | - |
| vB_VpaP_C2 ORF92 | + | 21828 | 21956 | 129 \| 42 | hypothetical protein | - | - | - | - | - |
| vB_VpaP_C2 ORF39 | + | 9401 | 9997 | 597 \| 198 | hypothetical protein | *Vibrio* phage vB_VpaS_OWB | YP_009948710.1 | 100% | 2.00E-81 | 98.54% |
| vB_VpaP_C2 ORF40 | + | 10199 | 11011 | 813 \| 270 | endolysin | *Vibrio* phage vB_VpaS_OWB | YP_009948710.1 | 100% | 5.00E-127 | 100.00% |
| vB_VpaP_C2 ORF42 | + | 12248 | 12673 | 426 \| 141 | holin | *Vibrio* phage vB_VpaS_OWB | YP_009948710.1 | 99% | 0 | 97.78% |
| vB_VpaP_C2 ORF56 | + | 25700 | 28378 | 2679 \| 892 | hypothetical protein | - | - | - | - | - |
| vB_VpaP_C2 ORF48 | + | 18353 | 18475 | 123 \| 40 | hypothetical protein | *Vibrio* phage vB_VpaS_OWB | YP_009948710.1 | 81% | 3.00E-82 | 99.16% |
| vB_VpaP_C2 ORF49 | + | 18743 | 18919 | 177 \| 58 | putative phosphoesterase | *Vibrio* phage vB_VpaP_KF2 | YP_009808068.1 | 100% | 3.00E-33 | 85.29% |
| vB_VpaP_C2 ORF50 | + | 19169 | 19261 | 93 \| 30 | hypothetical protein | *Vibrio* phage vB_VpaS_OWB | YP_009948710.1 | 100% | 0 | 99.88% |
| vB_VpaP_C2 ORF52 | + | 20693 | 20779 | 87 \| 28 | hypothetical protein | - | - | - | - | - |
| vB_VpaP_C2 ORF53 | + | 22022 | 22582 | 561 \| 186 | hypothetical protein | - | - | - | - | - |
| vB_VpaP_C2 ORF54 | + | 22592 | 24934 | 2343 \| 780 | hypothetical protein | - | - | - | - | - |
| vB_VpaP_C2 ORF55 | + | 24944 | 25690 | 747 \| 248 | hypothetical protein | - | - | - | - | - |
| vB_VpaP_C2 ORF57 | + | 29669 | 29800 | 132 \| 43 | hypothetical protein | *Vibrio* phage vB_VpaS_OWB | YP_009948710.1 | 100% | 8.00E-146 | 99.01% |
| vB_VpaP_C2 ORF58 | + | 30065 | 30172 | 108 \| 35 | DNA helicase | - | - | - | - | - |
| vB_VpaP_C2 ORF59 | + | 30335 | 30442 | 108 \| 35 | hypothetical protein | *Vibrio* phage BUCT233 | QWE49783.1 | 100% | 0 | 99.00% |
| vB_VpaP_C2 ORF62 | + | 37892 | 37984 | 93 \| 30 | hypothetical protein | *Vibrio* phage VP93 | YP_002875667.1 | 100% | 1.00E-07 | 47.27% |
| vB_VpaP_C2 ORF63 | + | 38000 | 38299 | 300 \| 99 | hypothetical protein | *Vibrio* phage vB_VpaP_KF1 | YP_009808040.1 | 100% | 3.00E-50 | 87.36% |
| vB_VpaP_C2 ORF64 | + | 38381 | 38608 | 228 \| 75 | hypothetical protein | *Vibrio* phage VP93 | YP_002875667.1 | 81% | 4.00E-66 | 95.15% |
| vB_VpaP_C2 ORF69 | + | 43271 | 43408 | 138 \| 45 | hypothetical protein | - | - | - | - | - |

| **Tabla S9. *V. parahaemolyticus* strains used in the study** | | | | | | | | |
| --- | --- | --- | --- | --- | --- | --- | --- | --- |
| **Strain** | **Date** | **Farm** | **Locality** | **Source** | **Latitude** | **Longitude** | **Accession** | **Organism** |
| M0605 | 22/07/2013 | CDM | Eldorado, Sinaloa | Shrimp stomach | 24.2056N | 107.2751W | SRR3167338 | *V. parahaemolyticus* M0605 |
| M0607 | 22/07/2013 | CDM | Eldorado, Sinaloa | Shrimp hepatopancreas | 24.2056N | 107.2751W | JAANII000000000 | *V. parahaemolyticus* M0607 |
| M0802 | 24/08/2013 | Aquastrat | Escuinapa, Sinaloa | Shrimp stomach | 22.4002N | 105.4921W | JAAIXL000000000 | *V. parahaemolyticus* M0802 |
| M0803 | 24/08/2013 | Aquastrat | Escuinapa, Sinaloa | Shrimp hepatopancreas | 22.4002N | 105.4921W | JAAIXK000000000 | *V. parahaemolyticus* M0803 |
| M0904 | 27/08/2013 | Aquastrat | Escuinapa, Sinaloa | Shrimp hepatopancreas | 22.4002N | 105.4921W | JAAIXM000000000 | *V. parahaemolyticus* M0904 |
| M0905 | 27/08/2013 | Aquastrat | Escuinapa, Sinaloa | Shrimp stomach | 22.4002N | 105.4921W | JAAKZP000000000 | *V. parahaemolyticus* M0905 |
| M2401 | 22/05/2019 | El Botetero | Angostura, Sinaloa | Shrimp hepatopancreas | 25.0540N | 108.0502W | JAAKZQ000000000 | *V. parahaemolyticus* M2401 |
| M2411 | 22/05/2019 | El Botetero | Angostura, Sinaloa | Shrimp hepatopancreas | 25.0540N | 108.0502W | JAAKZR000000000 | *V. parahaemolyticus* M2411 |
| M2413 | 22/05/2019 | El Botetero | Angostura, Sinaloa | Shrimp hepatopancreas | 25.0540N | 108.0502W | JAAKZS000000000 | *V. parahaemolyticus* M2413 |
| M2415 | 22/05/2019 | El Botetero | Angostura, Sinaloa | Shrimp hepatopancreas | 25.0540N | 108.0502W | JAAKZT000000000 | *V. parahaemolyticus* M2415 |

| **Table S10. Similarity of phages M3, C2, M83 and M9 with other reported phages** | | | |
| --- | --- | --- | --- |
| **Phage** | **Accession** | **Identidy (%)** | **Query Cover** |
| OWB | MN974282.1 | 97.55% | 97% |
| vB_VpaS_OWB | NC_048167.1 | 97.55% | 97% |
| vB_VpaP_KF1 | NC_048035.1 | 90.73% | 98% |
| vB_VpP_DE17 | MW250641.1 | 90.71% | 98% |
| vB_VpP_DE18 | MZ182247.1 | 90.59% | 99% |
| vB_VpP_FE11 | MT178448.1 | 90.13% | 98% |
| vB_Vc_SrVc2 | MW331544.1 | 91.45% | 96% |
| vB_Vc_SrVc9 | LR794124.1 | 91.45% | 96% |
| vB_VpP_DE10 | MZ592921.1 | 90.86% | 97% |
| vB_VpaP_MGD1 | MT501516.1 | 90.82% | 98% |
| vB_VpaP_KF2 | NC_048036.1 | 90.63% | 99% |
